# Extracellular vesicles: emerging anti-cancer drugs and advanced functionalization platforms for cancer therapy

**DOI:** 10.1080/10717544.2022.2104404

**Published:** 2022-08-01

**Authors:** Manling Wu, Min Wang, Haoyuan Jia, Peipei Wu

**Affiliations:** a Department of Clinical Laboratory, The First Affiliated Hospital of UST C, Division of Life Sciences and Medicine, University of Science and Technology of China, Hefei, Anhui, P.R. China; b Anhui Provincial Children’s Hospital, Hefei, Anhui, P.R. China; c Jiangsu Key Laboratory of Medical Science and Laboratory Medicine School of Medicine, Jiangsu University, Zhenjiang, Jiangsu, P.R. China; d Department of Clinical Laboratory, The Affiliated Wuxi People’s Hospital of Nanjing Medical University, Wuxi, Jiangsu, P.R. China

**Keywords:** Engineered EVs, cancer therapy, drug delivery, functionalization strategy, bioinspiration

## Abstract

Increasing evidences show that unmodified extracellular vesicles (EVs) derived from various cells can effectively inhibit the malignant progression of different types of tumors by delivering the bioactive molecules. Therefore, EVs are expected to be developed as emerging anticancer drugs. Meanwhile, unmodified EVs as an advanced and promising nanocarrier that is frequently used in targeted delivery therapeutic cargos and personalized reagents for the treatment and diagnosis of cancer. To improve the efficacy of EV-based treatments, researchers are trying to engineering EVs as an emerging nanomedicine translational therapy platform through biological, physical and chemical approaches, which can be broaden and altered to enhance their therapeutic capability. EVs loaded with therapeutic components such as tumor suppressor drugs, siRNAs, proteins, peptides, and conjugates exhibit significantly enhanced anti-tumor effects. Moreover, the design and preparation of tumor-targeted modified EVs greatly enhance the specificity and effectiveness of tumor therapy, and these strategies are expected to become novel ideas for tumor precision medicine. This review will focus on reviewing the latest research progress of functionalized EVs, clarifying the superior biological functions and powerful therapeutic potential of EVs, for researchers to explore new design concepts based on EVs and build next-generation nanomedicine therapeutic platforms.

## 1. Introduction

Cancer is one of the most significant burdens that a human could experience all over the world (Torre et al., 2015). In particular, with the change of disease patterns and the trend of population aging, cancer prevention and treatment are facing a grim situation. During the past several decades, despite years of investigations, the scientists still have not developed effective therapies for many types of tumors. Currently, lacks of specificity and effectiveness remain the main limitations of clinical tumor therapy. Therefore, there is an urgent need to find new therapeutic regimens for the precise treatment of tumors. EVs are nanoscale to micron-sized membrane structure vesicles with a size ranging from 30 to 5000 nm (Cocozza et al., 2020), which are shed from cell membranes or secreted by almost all bacteria, archaea and eukaryotic cells in a constitutive or fine-tuning manner (van Niel et al., 2018). EVs are spherical, bilayered proteolipids that harbor multiple biomolecules including proteins, nucleic acids, lipids and metabolites. The interaction between tumor cells and tumor microenvironment is closely related to tumor initiation, development, metastasis and drug resistance. As one of the important components of tumor microenvironment, EVs play an important role in tumor relapse and drug resistance, angiogenesis, immune monitoring and other aspects. In addition, EVs are also the main content of tumor liquid biopsy, which can provide new insights for the diagnosis, treatment and prognosis of tumor.

EVs do not only play critical roles in numerous pathophysiological processes, but also have broad clinical application prospects in the diagnosis, prognosis and therapy of cancer (Yang et al., 2019; Pan et al., 2021). Currently, many studies have shown that EVs have a double-edged sword effect, which can promote and inhibit tumor under different conditions. However, the pro-tumor and anti-tumor functions of EVs are closely related to the origin of parental cells. EVs released by tumor cells and cells in their microenvironment almost promote tumor initiation, development, metastasis, immune escape and drug resistance. However, numerous studies shown that unmodified EVs are employed as advanced therapeutic ingredient for treating various common and refractory tumor diseases, including cancer (Wiklander et al., 2019). For example, dendritic cells (DCs) and tumor cell-derived EVs can be also used as bioactive reagent to enhance anti-tumor immune response of the body (Tan et al., 2010). In addition, stem cell therapy and tumor bio-immunotherapy are gradually becoming an emerging and efficient antitumor therapy manner after surgery, radiotherapy and chemotherapy. Recent studies demonstrated that mesenchymal stem cells (MSCs) derived EVs as a key component of cell paracrine have emerged as potential therapeutic agents that exert their antitumor effects. Proteins, miRNAs, and other antitumor active molecules carried by MSC-EVs can inhibit growth and retard tumor progression by inhibiting the proliferation, migration, invasion and epithelial-mesenchymal transition (EMT) of tumor cells.

Unmodified EVs possess excellent biocompatibility, higher safety and better physiochemical stability, as well as good antitumor effects. However, it still has some drawback including low yield, insufficient targeting and circulation instability. Currently, researchers are trying to develop more efficient and convenient methods to enhance the therapeutic applications of EVs in cancer therapy (Zhang et al., 2021). Therefore, engineered EVs are attracting wide interest of biomedical workers and becoming a new alternative cell-free strategy for cancer therapy. In this review, we generalize the research status of current methods for separation, identification and functionalization of EVs and discuss the potential application of engineered EVs in cancer therapy.

## 2. Biogenesis, release, uptake and components of EVs

**Figure 1.**
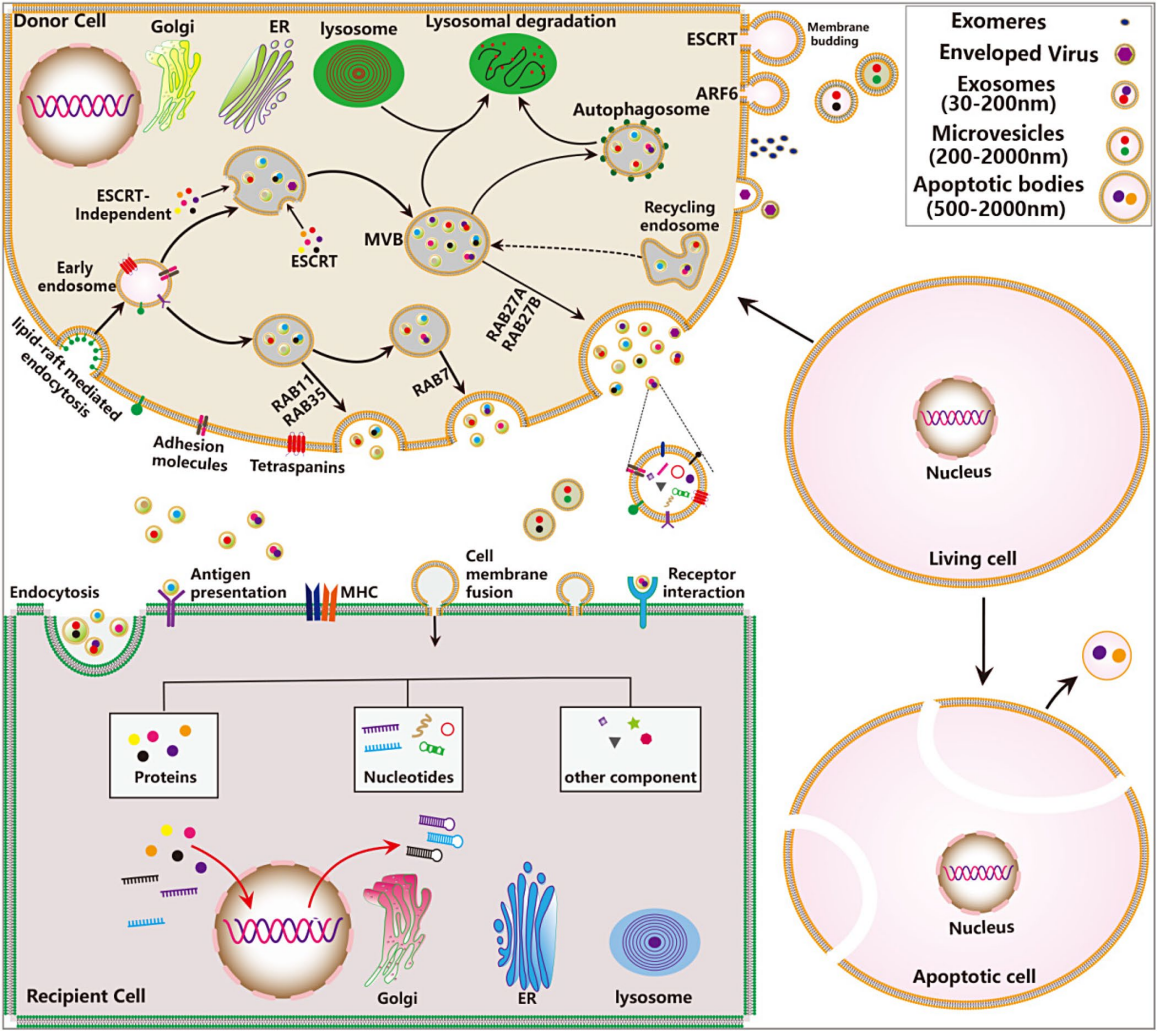
The biogenesis, release and uptake of EVs and their interactions with target cells. The biosynthesis and regulatory mechanisms of different types of secreted EVs. Three major mechanisms have been suggested to mediate the uptake of EVs, including cell membrane fusion, receptor-ligand interactions and endocytosis.

EVs are a general term for subsets of membrane structures, which are secreted by almost all bacteria, archaea and eukaryotic cells in a constitutive or regulated manner. The EVpedia (http://evpedia.info), a complete and comprehensive database of proteomics, transcriptomics and metabolomics for the systematic analyses of EVs, contains a variety of EVs from archaea, bacteria, and eukaryotes, including humans. Prokaryotes and eukaryotes release different vesicles into the extracellular space, these various types of secreted nanosized membrane vesicles with different size, density, composition and intracellular sources. EVs have diverse nomenclature, and the EVpedia provides a complete overview of vesicles nomenclature and classification. EVs contain many subtypes, including enveloped virus, exosomes, ectosomes, microvesicles, microparticles, large oncosomes, and apoptotic bodies. The latest research found that exosomes have different subtypes, which are named small exosomes (Exo-S, 60–80 nm) and large exosomes (Exo-L, 90–120 nm) respectively. These exosomes subsets have different biophysical and molecular characteristics. Meanwhile, the authors also discover a previously unknown class of nanoparticles, called exomere (30–50 nm). Compared with exosomes, exomere is a discrete and abnormally small nanoparticle with a diameter of about 35 nm that exhibits different proteins, lipids, RNAs and DNAs profiles (Kowal et al., 2016; Zhang et al., 2018; Hoshino et al., 2020). EVs are a highly heterogeneous population. Due to the lack of universal molecular markers to distinguish different types of EVs, the nomenclature and classification of vesicles are still complex and controversial. To resolve these controversies and further standardize the study of EVs, the ISEV publishes its latest guidelines and recommends using the physical and chemical properties to describe EVs (Thery et al., 2018). According to EVs notable differences in physical properties, biogenesis process and functions, these vesicles are classified into three major categories: exosomes, microvesicles and apoptotic bodies (Figure 1).

EVs are secreted by almost all types of cells in pathophysiological conditions and are found in a variety of human body fluids such as blood, urine, and ascites, which can mediate cellular communication and reflect their cell of origin. Exosome formation is a continuous fine-tuning process, which are divided into the following three stages: endocytosis, multivesicular bodies (MVBs) formation, fusion and efflux. Firstly, cells produce endocytic vesicles through lipid-mediated endocytosis, multiple endocytic vesicles fuse to form the early endosome. The early endosome again invaginate and encapsulates the intracellular material form the intraluminal vesicles (ILVs), and gradually evolving into late endosomes. Finally, MVBs directly fuse with the plasma membrane and release ILVs into the extracellular space known as exosomes. Other EVs (including apoptotic bodies, microvesicles, ectosomes, microparticles, and large oncosomes) can be generated directly from the plasma membrane by outward membrane budding manner (Cocozza et al., 2020). Currently, researches show that the formation of microvesicles is related to the asymmetric distribution of phospholipids in the cell membrane bilayer (Hugel et al., 2005).

**Figure 2.**
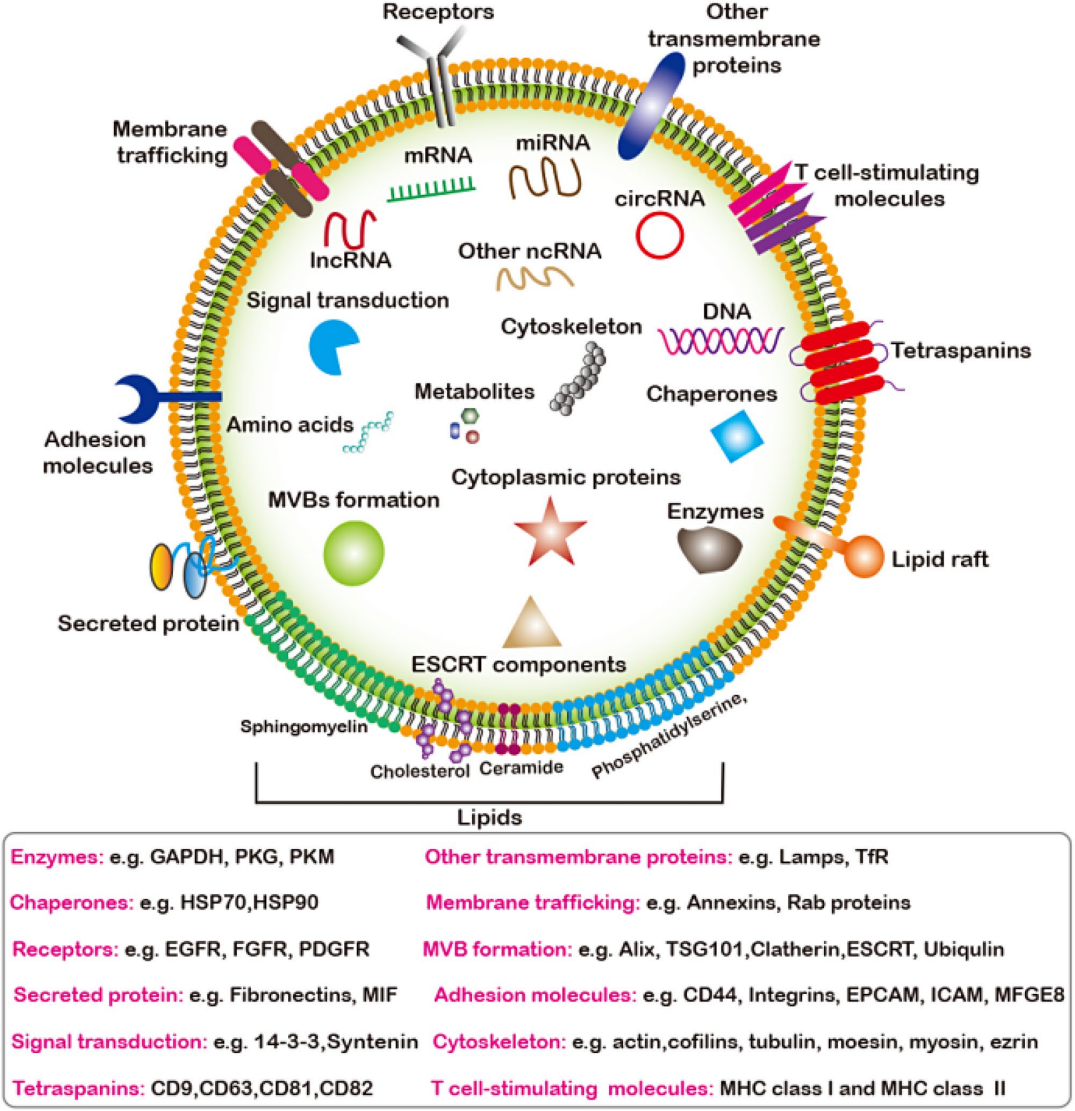
The overall compisition of EVs. The main components of EVs include nucleic acids, proteins, lipids and metabolites. Main classification of nucleic acids and proteins components enriched in EVs that mediates the intercellular communication between different cell types in the body, thus affecting the normal and pathological stateand their potential biological functions.

Both the endosomal sorting complex required for transport (ESCRT)-dependent and independent pathways are responsible for the sorting of cargos into the MVBs and participate in regulating exosomes release (He et al., 2018). Multiple regulatory molecules (including proteins, nucleic acids, and lipids) and pathway have been found to critically regulate exosome secretion, such as the Rab guanosine triphosphatases (GTPases) (Colombo et al., 2014), RAL GTPases (Hyenne et al., 2015), vacuolar protein sorting protein 33 b (VPS33B) (Gu et al., 2016), heparinase (Thompson et al., 2013), lncRNA HOTAIR (Yang et al., 2019), ceramides (Trajkovic et al., 2008), phospholipase D and phosphatidic acid, as well as sphingolipids (Egea-Jimenez & Zimmermann, 2018; Verderio et al., 2018), oncogenes and tumor suppressors (Yu et al., 2005), and p53-regulated protein tumor suppressor-activated pathway 6 (TSAP6) (Lespagnol et al., 2008). Recently, some new regulatory moleculessuch as kidney and brain expressed protein (KIBRA) (Song et al., 2019), ISGylation of TSG101 (Villarroya-Beltri et al., 2016), mTORC1 (Zou et al., 2019), sirtuin1 (SIRT1) (Latifkar et al., 2019; Li et al., 2019) are revealed. In addition, intracellular and intercellular microenvironmental changes also have important effects on exosome secretion, such as microenvironmental pH (Parolini et al., 2009), intracellular Ca2^+^ (Savina et al., 2003), glucose deprivation and lack of oxygen (Mulcahy et al., 2014). The donor cells derived EVs transfer genetic cargos to the receptor cells through the following three main ways: (1) endocytosis; (2) receptor-ligand interaction; (3) direct fusion with the plasma membrane (Figure 1) (Kalluri & LeBleu, 2020). The type of exosomes and the type of recipient cells also influence the target cells uptake exosomal cargos. EVs contain diverse subsets of proteins, nucleic acids, lipids, metabolites (Figure 2).

## 3. Unmodified EVs for cancer therapy

### 3.1. Stem cell-derived EVs

**Figure 3.**
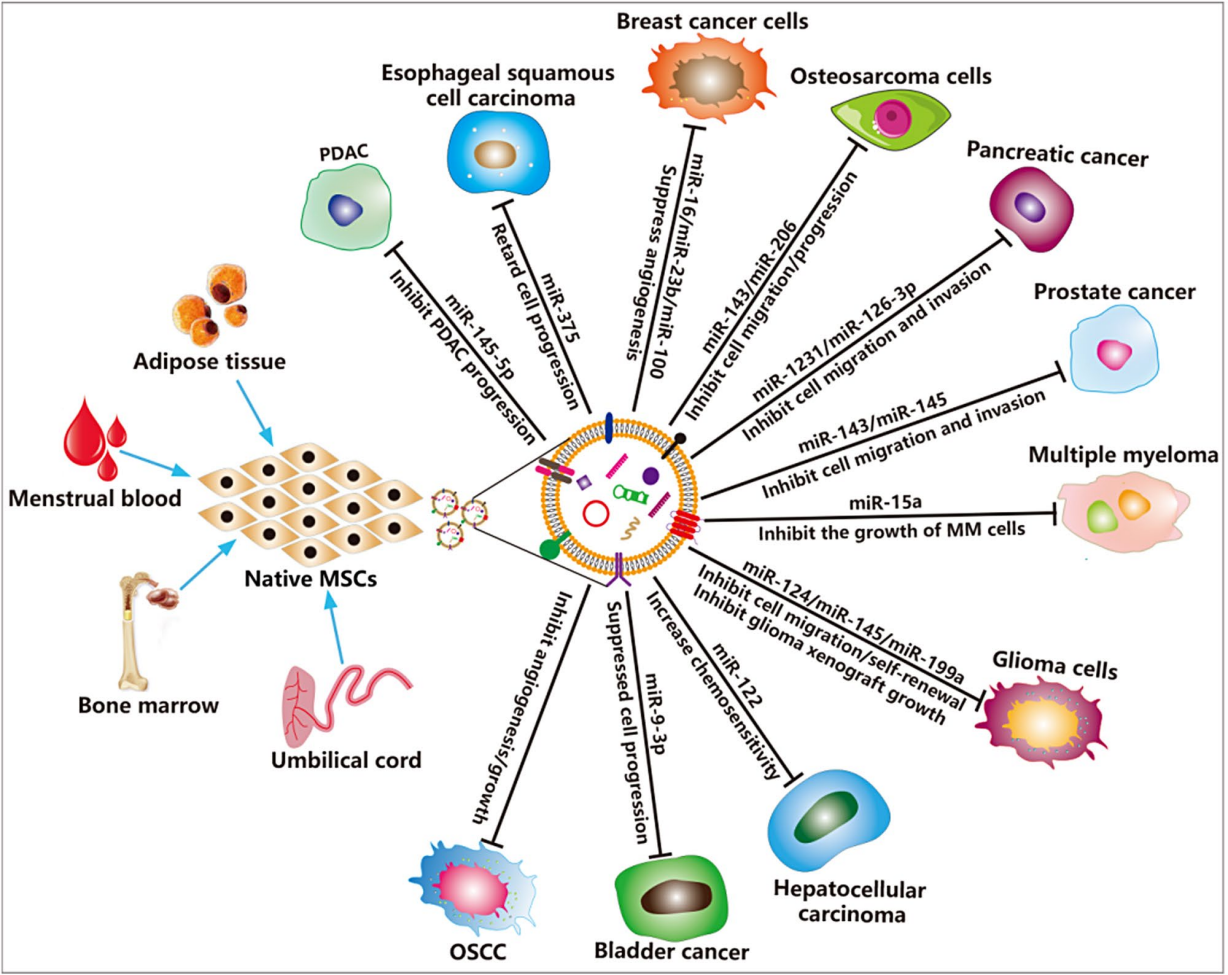
MSCs derived EVs for cancer therapy. The EVs derived from MSCs carry a variety of bioactive molecules that play an effective anti-tumor efficacy in various cancer diseases. EVs derived from MSCs are also ideal candidates for engineered EVs.

MSCs are a type of undifferentiated adult stem cells that derived from the mesoderm, they possess high self-renewal and multidirectional differentiation potential, and can differentiate into multiple mesenchymal tissues (Hass et al., 2011). MSCs are abundant in almost all human tissues and organs, such as bone marrow, umbilical cord blood, placenta, adipose tissues (Heo et al., 2016), umbilical cord (Beeravolu et al., 2017), dental pulp (Liu et al., 2018), menstrual blood (Xiang et al., 2017; Rosenberger et al., 2019), urine (Bento et al., 2020), chorionic and amniotic membrane (Chen et al., 2019). Currently, the mounting evidence revealed that MSCs are crucial components of the tumor microenvironment and can inhibit tumor progression in different types of tumors (Ridge et al., 2017; Weng et al., 2021). Several studies revealed that EVs derived from native embryonic stem cells (ESCs) (Zhu et al., 2019) and other MSCs (Zhao et al., 2020) can suppress various tumors progression via delivering tumor-suppressive miRNAs into cancer cells, thereby possibly enabling a clinically relevant EV-based theraoeutic strategy for complex refractory tumor diseases (Table 1 and Figure 3). Human bone marrow MSCs (hBMMSCs) and human menstrual MSCs (hMMSCs) derived EVs can inhibit different tumor malignant progression (Bruno et al., 2013), and restrain angiogenesis and tumor growth of oral squamous cell carcinoma (Rosenberger et al., 2019), respectively. In addition, human umbilical cord MSCs (hucMSCs) also enhanced the systemic efficacy of radiotherapy-induced cell death in tumor and metastatic tumor foci (de Araujo Farias et al., 2018). However, the specific mechanism of these MSCs anti-tumor effect is not clear.

**Table 1.** The application of stem cells derived unmodified and functionalized EVs in cancer therapy.

EVs type	EVs sources	Guest molecules	Target gene	Applications	Ref
sEVs	MenSCs, hucMSCs	—	VEGF	Inhibit angiogenesis and tumor growth of OSCC	(Rosenberger et al., 2019)
MVs	BMMSCs	—	—	Inhibit tumor growth	(Bruno et al., 2013)
sEVs	BMMSCs	miR-206	TRA2B	Inhibit osteosarcoma progression	(Zhang et al., 2020)
sEVs	mBMMSCs	miRNA-9-3p	ESM1	Inhibit bladder cancer growth	(Cai et al., 2019)
sEVs	hBMMSCs	miR-100	mTOR/HIF-1α/VEGF	Suppress angiogenesis in breast cancer cells	(Pakravan et al., 2017)
sEVs	mBMMSCs	miR-16	VEGF	Suppress angiogenesis in breast cancer cells	(Lee et al., 2013)
sEVs	hBMMSCs	miR-23b	MARCKS	Promote dormancy in metastatic breast cancer cells	(Ono et al., 2014)
sEVs	hucMSCs	—	Activated caspase pathway	Enhance imatinib-induced apoptosis in leukemia cells	(Liu et al., 2018)
Apo EVs	mBMMSCs	—	—	Ameliorate multiple myeloma by activating Fas/FasL pathway	(Wang et al., 2021)
EVs	GM-CSF-hESCs	—	—	A preventive vaccine against cancer	(Yaddanapudi et al., 2019)
sEVs	BMMSCs	miR-15a	—	Inhibit the growth of MM cells	(Roccaro et al., 2013)
sEVs	GA-hMSCs	miR-1587	NCOR1	Enhance the aggressiveness of glioblastoma	(Figueroa et al., 2017)
sEVs	hBMMSCs	miR-199a	AGAP2	Inhibit glioma progression	(Yu et al., 2019)
sEVs	hADMSCs	antagomiR-222/223	—	Promote early dormancy of breast cancer	(Bliss et al., 2016)
sEVs	hADMSCs	miR-122	CCNG1, ADAM10, IGF1R	Increase chemosensitivity of hepatocellular carcinoma	(Lou et al., 2015)
sEVs	hucMSCs	miR-145-5p	Smad3	Inhibit PDAC progression	(Ding et al., 2019)
sEVs	hBMMSCs	miR-143	TFF3	Inhibit prostate cancer growth	(Che et al., 2019)
sEVs	MSCs	miR-124, miR-145	SCP-1, Sox2	Inhibit gliom cells growth	(Lee et al., 2013)
sEVs	hBMMSCs	miR-143	—	Inhibit osteosarcoma cells migration	(Shimbo et al., 2014)
sEVs	hucMSCs	miR-375	ENAH	Retard ESCC progression	(He et al., 2020)
sEVs	hBMMSCs	PTX	—	For breast cancer chemotherapy	(Kalimuthu et al., 2018)
sEVs	MSCs	siRNA	PLK-1	Induced cell apoptosis and necrosis	(Greco et al., 2016)
sEVs	MSCs	miR-146b	EGFR	Inhibit glioma xenograft growth	(Katakowski et al., 2013)
sEVs	hBMMSCs	miRNA-1231	—	Inhibit the activity of pancreatic cancer	(Shang et al., 2019)
sEVs	ADMSCs	miR-145	—	Suppress prostate cancer progression	(Takahara et al., 2016)
sEVs	BMMSCs	miR-126-3p	ADAM9	Inhibit pancreatic cancer development	(Wu et al., 2019)
sEVs	mBMMSCs	miR-133b	EZH2	Suppress glioma progression	(Xu et al., 2019)
sEVs	hBMMSCs	anti-miR-9	P-gp	Reverse chemoresistance	(Munoz et al., 2013)
sEVs	hMSCs	SPIONs	—	Target tumor cell ablation	(Altanerova et al., 2017)
sEVs	hBMMSCs	Circ0030167	—	Inhibit the malignant progression of pancreatic cancer	(Yao et al., 2021)

Some studies demonstrated that miRNAs are enriched in MSCs derived EVs are responsible for their anti-tumor effects. For example, BMMSC-EVs containing miR-206 inhibits osteosarcoma progression by targeting transformer 2β (TRA2B) (Zhang et al., 2020), and containing miR-9-3p inhibits the growth and metastasis of bladder cancer by targeting ESM1 (Endothelial cell specific molecule 1) gene expression (Cai et al., 2019). Moreover, miR-100 and miR-16 shuttled by BMMSC-EVs suppressed breast cancer cells angiogenesis *in vitro* through modulating the mTOR/HIF-1α/VEGF signaling axis (Pakravan et al., 2017), suppressed angiogenesis by down-regulating VEGF expression in breast cancer cells (Lee et al., 2013), respectively. Again, BMMSC-EVs containing miR-23b promoted dormancy in metastatic breast cancer cells by targeting MARCKS (Myristoylated alanine-rich C-kinase substrate) (Ono et al., 2014). Adipose MSCs (ADMSCs) derived EVs containing miR-145 increased the expression of Caspase 3/7 and reduced anti-apoptotic protein Bcl-xL expression in prostate cancer cells (Takahara et al., 2016). HucMSC-EVs enhanced the sensitivity of human leukemia cells K562 to imatinib-induced apoptosis via activation of caspase signaling pathway (Liu et al., 2018). Unlike normal MSC-derived EVs, the role of apoptotic MSC-derived EVs in tumors is unclear. Recently, Wang et al. demonstrated that staurosporine-induced apoptotic murine BMMSCs derived EVs (apoEVs) induce multiple myeloma (MM) cell apoptosis and inhibit MM cell growth by activating Fas/FasL mediated apoptotic signaling pathways (Wang et al., 2021). Yaddanapudi et al. reported that GM-CSF expressing human ESCs release EVs is an effective prophylactic vaccine for cancer prevention (Yaddanapudi et al., 2019). However, the application of EVs derived from MSCs in tumors is still controversial. Studies have also shown that MSC-derived EVs can promote the malignant progression of tumors, which may be closely related to the state of the stem cells themselves, the environment in which they are located, and the isolation process of EVs (Zhu et al., 2012; Roccaro et al., 2013; Figueroa et al., 2017; Zhao et al., 2020).

### 3.2. Tumor cell-derived EVs

Tumor bio-immunotherapy can mobilize the immunity of human body, antitumor immune response. By reinfusion of autologous immune cells, such as DCs, T lymphocytes, natural killer (NK) cells, and tumor cell lysates, the immune system can be mobilized cells to stimulate and enhance the antitumor immune response, and thus inhibit tumor growth, metastasis and recurrence (Zhang et al., 2021). Tumor bioimmunity, as another emerging antitumor therapy technology, has more extensive application prospects (Yang, 2015). Whole tumor cell lysates have been implemented as tumor antigens for cancer vaccine development, and EVs derived from tumor cells (T-EVs) are also facing vaccine transformation applications because they have similar functions to cells (Table 2). T-EVs have a double-edged sword effects in tumors. TEVs stimulate T cell activation (Yao et al., 2014) and trigger its mediated anti-tumor immune response, thereby inhibiting tumor growth and metastasis (Wolfers et al., 2001; Lee et al., 2012; Gu et al., 2015; Wang et al., 2020). However, cargos carried by TEVs can promote tumor occurrence, development, metastasis, resistance and immune escape. Therefore, the direct use of T-EVs for cancer treatment has huge safety risks, which prevents them from becoming safe cellular cancer vaccines. In phase I clinical trial, autologous ascites-derived EVs combined with granulocyte-macrophage colony stimulating factor (GM-CSF) is feasible and safe immunotherapy for colorectal cancer treatment (Wolfers et al., 2001). In addition, TEVs delivered immunostimulatory CpG DNA enhances anti-tumor immune activity (Morishita et al., 2016). PEGylated tumor cell membrane derived NVs combined with anti-programmed death-1 (αPD-1) IgG as a new vaccine platform for cancer immunotherapy (Ochyl et al., 2018). Although T-EVs show beneficial anticancer immune effects, but there is still a need to explore more measures to avoid its potential clinical application risks.

**Table 2.** The application of other cells derived EVs in cancer therapy.

EVs type	EVs sources	Guest molecules	Target gene	Applications	Ref
sEVs	K562 cells	—	—	Induce anti-leukemic immunities	(Yao et al., 2014)
sEVs	Vδ2-T cells	—	—	Induce antitumor immunity	(Wang et al., 2020)
NVs	Autologous tumor	—	—	Inhibit melanoma growth and metastasis	(Lee et al., 2012)
sEVs	Tumor cells	—	—	Induce CD8+ T-cell-dependent antitumor effects	(Wolfers et al., 2001)
sEVs	Tumor cells	CpG DNA	—	For cancer immunotherapy	(Morishita et al., 2016)
sEVs	PEGylated Tumor cells	—	—	For cancer immunotherapy	(Ochyl et al., 2018)
sEVs	DC cells	—	—	Kill tumor and activate NK cells	(Munich et al., 2012)
sEVs	DC cells	—	—	Maintenance immunotherapy	(Besse et al., 2016)
sEVs	AFP express DC cells	—	—	For HCC immunotherapy	(Lu et al., 2017)
sEVs	DC cells	Fluorouracil	—	Enhance anti-colon cancer effect	(Xu et al., 2020)
NVs	M1 Macrophage	aPD-L1	—	Potentiate aPD-L1 anticancer efficacy	(Choo et al., 2018)
EVs	M2 macrophage	miRNAs	—	Inhibit cell migration and invasion of gliomas	(Yao et al., 2021)
sEVs	NK cells	—	—	Target and therapy of glioblastoma	(Zhu et al., 2018)
sEVs	NK cells	—	—	Exert therapeutic effect in melanoma	(Zhu et al., 2017)
sEVs	NK cells	—	—	Antitumor activity of cytokine-activated NK cells	(Shoae-Hassani et al., 2017)
sEVs	NK cells	—	—	Maintain immune surveillance and homeostasis	(Lugini et al., 2012)
sEVs	NK cells	miR-186	MYCN AURKATGFBR1TGFBR2	Inhibit neuroblastoma growth and immune escape	(Neviani et al., 2019)
sEVs	NK cells	miR-3607-3p	IL-26	Inhibited pancreatic cancer progression	(Sun et al., 2020)
EVs	IL15-cultured NK cells	—	—	Enhance the anti-tumor effect	(Zhu et al., 2019)
sEVs	IL2/IL15-NK cells	DNAM1	—	Mediate cytotoxicity of tumor	(Di Pace et al., 2020)
sEVs	Circulating NK cells	—	—	Exhibit antitumoral activity	(Kang et al., 2021)
sEVs	CD4+ T cells	—	—	Inhibit CD8^+^ T cells responses and anti-tumor immunity	(Zhang et al., 2011)
EVs	Activated CD8^+^ T cells	—	—	Prevent tumor progression	(Seo et al., 2018)
sEVs	Activated T cells	PD-1	—	Attenuate PD-L1-induced immune dysfunction	(Qiu et al., 2021)
sEVs	CD45RO^-^CD8^+^ T cells	miR-765	—	Restrict cancer development	(Zhou et al., 2021)
NVs	Activated CD8^+^ T cells	Granzyme B, PD-1,	—	For cancer immunotherapy	(Hong et al., 2021)
NVs	Ginseng	—	—	Inhibit melanoma growth	(Cao et al., 2019)
NVs	Citrus limon	—	—	Inhibit CML xenograft growth	(Raimondo et al., 2015)
NVs	PEGylated asparagus cochinchinensis	—	—	Inhibit tumor growth	(Zhang et al., 2021)

### 3.3. DC-derived EVs

**Figure 4.**
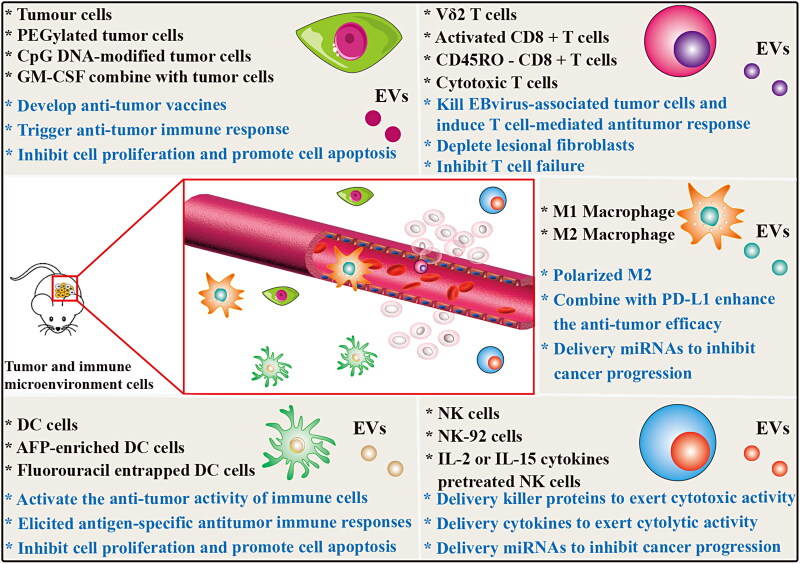
Other Mammalian cells derived EVs for cancer therapy. The EVs derived from tumor cells and main immune cells carry a variety of bioactive molecules that play an effective anti-tumor efficacy in various cancer diseases. EVs derived from tumor cells and DC cells mainly induce specific humoral and cellular immune responses in the body, enhance the anticancer ability of the body and prevent the growth of tumors. M1-type macrophages are derived from EVs polarized M2-type macrophages into anti-tumor M1-type macrophages and release inflammatory cytokines to play a role in tumor therapy. EVs from NK cells and T cells play a therapeutic role by releasing tumor-killing molecules and cytotoxic effects.

DCs are the most powerful antigen-presenting cells. Mature DCs express high levels of co-stimulatory molecules, adhesion molecules, functional MHC peptide complexes, and other components that interact with immune cells. However, immature DCs expressed low levels of co-stimulators and adhesion factors, but had strong antigen-phagocytosis ability. DC-derived EVs also express these functional molecules with antigen-presenting ability, and promote the potential of immune cell-dependent tumor rejection (Figure 4). Currently, phase I and II clinical trials based on DC-derived EVs have been conducted in advanced malignancies, demonstrating the feasibility and safety of this approach (Pitt et al., 2016). DC-derived EVs can activate the anti-tumor activity of NK cells (Munich et al., 2012) and T cells (Besse et al., 2016) via TNF superfamily ligands and IFN-γ, respectively. Lu et al. found that α-fetoprotein (AFP)-enriched DC-derived EVs elicited strong antigen-specific antitumor immune responses and retarded tumor growth in ectopic, orthotopic and carcinogen-induced HCC mice (Lu et al., 2017). Fluorouracil entrapped DC-derived EVs can significantly inhibit the proliferation of colon cancer cells and induce their apoptosis, exerting an anti-tumor effect (Xu et al., 2020). In recent years, it has been confirmed that the engineered DC-derived EVs exhibit enhanced anti-tumor efficacy and is most likely to be the first vaccine to be used in clinical transformation.

### 3.4. Macrophage-derived EVs

Tumor-associated macrophages (TAM) are the most common in tumor microenvironment, accounting for about 50% of the tumor microenvironment. Macrophages affected by tumor microenvironmental cytokines can differentiate into different types of TAM, which are mainly divided into tumor suppressor M1 type and tumor promote M2 type according to their functions. M1 polarized macrophage-derived nanovesicles (NVs) can repolarize M2 TAMs to M1 macrophages that release pro-inflammatory cytokines and induce antitumor immune responses (Figure 4). Moreover, M1 macrophage-derived NVs combined with immune checkpoint inhibitor PD-L1 enhanced the anti-tumor efficacy (Choo et al., 2018). Glioma originated from glial cells and is the most common primary brain tumor, accounting for about 30–40% of all intracranial tumors. M2 macrophage-derived EVs promote the migration and invasion of glioma cells, while the low-expressed miR-15a and miR-92a in EVs have opposite effects on glioma cells. The miR-15a and miR-92a contained in M2 macrophage-derived EVs inhibit the migration and invasion of glioma cells through CCND1 and RAP1B activation of the PI3K/AKT/mTOR signaling pathway, respectively (Yao et al., 2021).

### 3.5. NK cell-derived EVs

NK cells originated from hematopoietic stem cells, which have dual roles of effector and regulatory cells in innate immunity. NK cells can inhibit the malignant biological behavior of tumors through their direct cytolytic activity, and cytokine secretion. In recent studies, EVs, as a key component of NK cell therapy, have been proven to be effective in cancer therapy (Zhu et al., 2018). EVs derived from NK cells exert tumor cell killing and inhibitory functions through four main mechanisms, including Fas/Fas ligand (FasL) pathway, perforin/granzyme pathway, tumor necrosis factor (TNF)-α pathway, and miRNA-mediated targeted regulation pathways (Figure 4). Several studies have confirmed that NK cells derived EVs containing killer proteins such as FasL and perforin, and these molecules could activate immune cells and display cytotoxic activity in several tumor cell lines (Lugini et al., 2012; Shoae-Hassani et al., 2017; Zhu et al., 2017). In addition, Zhu et al. also confirmed for the first time NK-92 cells derived EVs contain TNF-α, which affected the melanoma cell proliferation, survival, and apoptosis (Zhu et al., 2017). Neviani et al. found that NK cell derived EV miR-186 inhibits neuroblastoma growth and immune escape by targeting MYCN, AURKA, TGFΒR1 and TGFΒR2 (Neviani et al., 2019). Furthermore, Sun et al. found that NK cells derived EV miR-3607-3p inhibits pancreatic cancer progression by targeting tumor-promoting cytokines IL-26 (Sun et al., 2020). Interestingly, using IL-2 or IL-15 cytokines to stimulate NK cells can significantly enhance the anti-tumor ability of their derived EVs (Zhu et al., 2019; Di Pace et al., 2020). Mechanically, Di Pace et al. found that IL-2 or IL-15-stimulated NK cells derived EVs exert their cytolytic effect against tumor by delivering IFN-γ, lymphocyte function-associated antigen-1 (LFA-1), DNAX accessory molecule-1 (DNAM1) and programmed cell death protein (PD-1) (Di Pace et al., 2020). Recently, Kang et al. designed a novel microfluidic system to collect non-small cell lung cancer patient-specific NK cells and on-chip biogenesis of circulating NK derived EVs. The development of this high-throughput, general-purpose device for the acquisition of NK derived EVs has demonstrated cytotoxic effects on CTC, and thus may hold a promise for antitumor therapies based on autologous patient EVs (Kang et al., 2021). These findings suggest that unmodified or functionalized NK cell-derived EVs are expected to become a new and powerful strategy for anti-tumor immunotherapy.

### 3.6. T cell-derived EVs

Helper T cells and cytotoxic T cells release their EVs to mediate interactions and functional changes (Figure 4). For example, activated ovalbumin (OVA)-specific CD4^+^ T cell released EVs inhibit CD8^+^ cytotoxic T-lymphocyte responses and antitumor immunity (Zhang et al., 2011). This study shows that CD4^+^ T cell-EVs are expected to be potential therapeutic agents for autoimmune-related diseases. However, CD8 ^+^ T cell EVs have been found to be beneficial for tumor immunotherapy. Activated CD8 ^+^ T cell from healthy mice release cytotoxic EVs prevent tumor invasion and metastasis by depleting lesional fibroblastic stroma cells (Seo et al., 2018). Wang et al. found that Vδ2-T cells derived EVs enriched in FasL, TRAIL, NKG2D, CD80, CD86, MHC class I and II, and these components can directly kill Epstein-Barr virus-associated tumor cells and induced T cell-mediated antitumor response (Wang et al., 2020). These findings indicate that T cell-derived EVs may be a promising strategy for tumor therapy. Currently, there exists few studies on the use of T cell derived EVs for direct anti-tumor or as tumor drug delivery vehicles. Recently, Qiu et al. found that activated T cell-derived exosomal PD-1 can induce cell surface or exosomal PD-L1 internalization, and thereby restoring tumor surveillance and attenuating PD-L1-induced immune dysfunction in triple-negative breast cancer (Qiu et al., 2021). CD45RO^-^ CD8^+^ T cell-derived EVs released miRNAs to restrict estrogen-driven endometrial cancer development via regulation of the ERβ/miR-765/PLP2/Notch signaling axis (Zhou et al., 2021). NVs from cytotoxic T cells produced by continuous extrusion of micro/nanopore membranes, inhibited T cell failure, and exhibited superior antitumor activity in the immunosuppressed tumor microenvironment (Hong et al., 2021).

### 3.7. Plant cell-derived EVs

Ginseng-derived EVs inhibited the growth of melanoma by changing the polarization of macrophages (Cao et al., 2019). Citrus limon-derived EVs inhibited cancer cell proliferation in different tumor cell lines and suppress CML xenograft growth by activating a TRAIL-mediated apoptotic cell death (Raimondo et al., 2015). EVs derived from plants provide a promising nanoplatform that can be applied to anti-tumor therapy with negligible side effects. PEGylated Asparagus cochinchinensis derived EV-like NVs could significantly inhibited tumor growth in Hep G2 cell xenograft model (Zhang et al., 2021).

## 4. Functionalized EVs for cancer therapy

**Figure 5.**
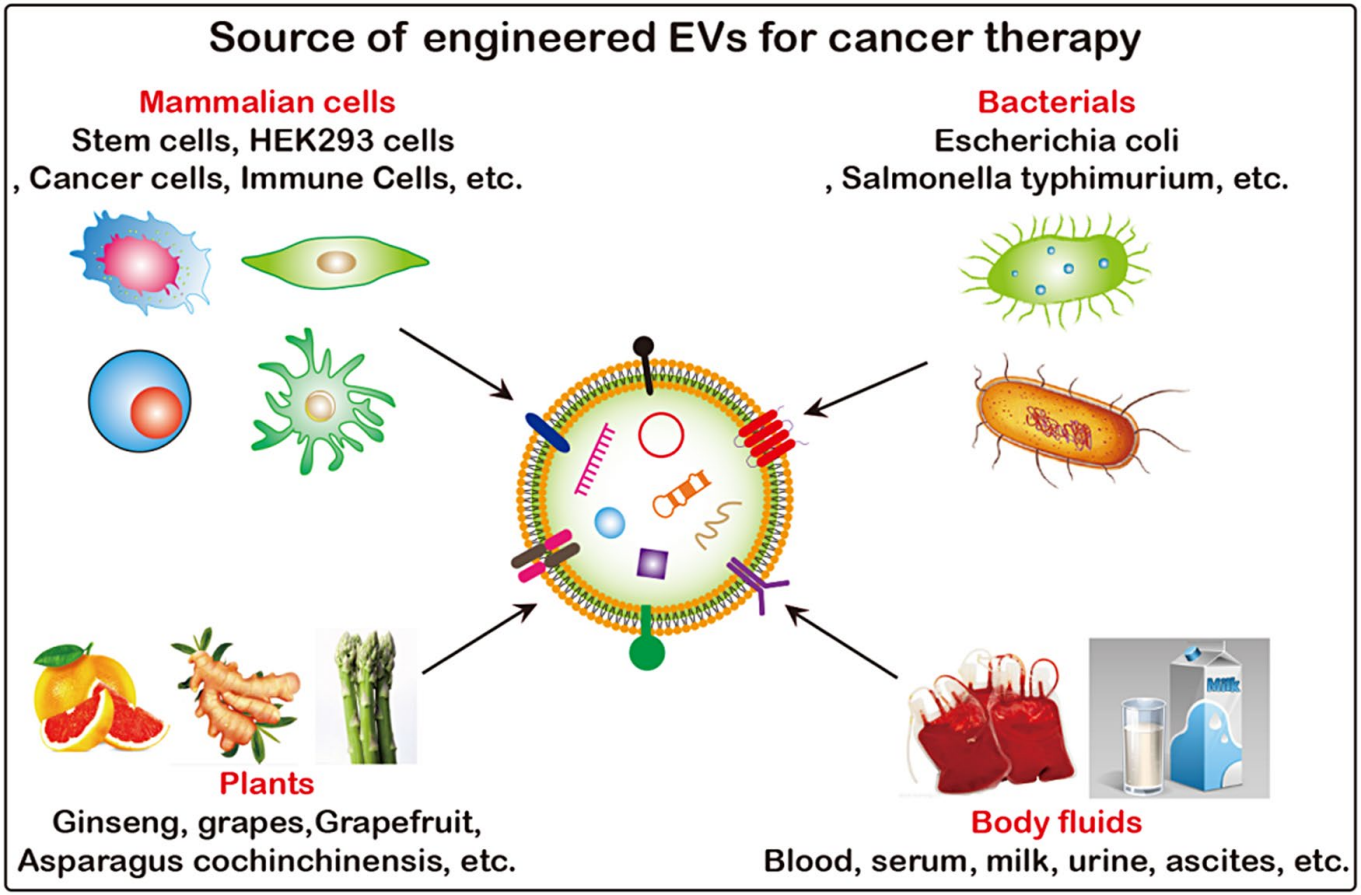
Source of functionalized EVs. EVs can be produced by almost all prokaryotic and eukaryotic, and plant cells, and are widely present in body fluids such as blood, urine, ascites and milk, etc.

**Figure 6.**
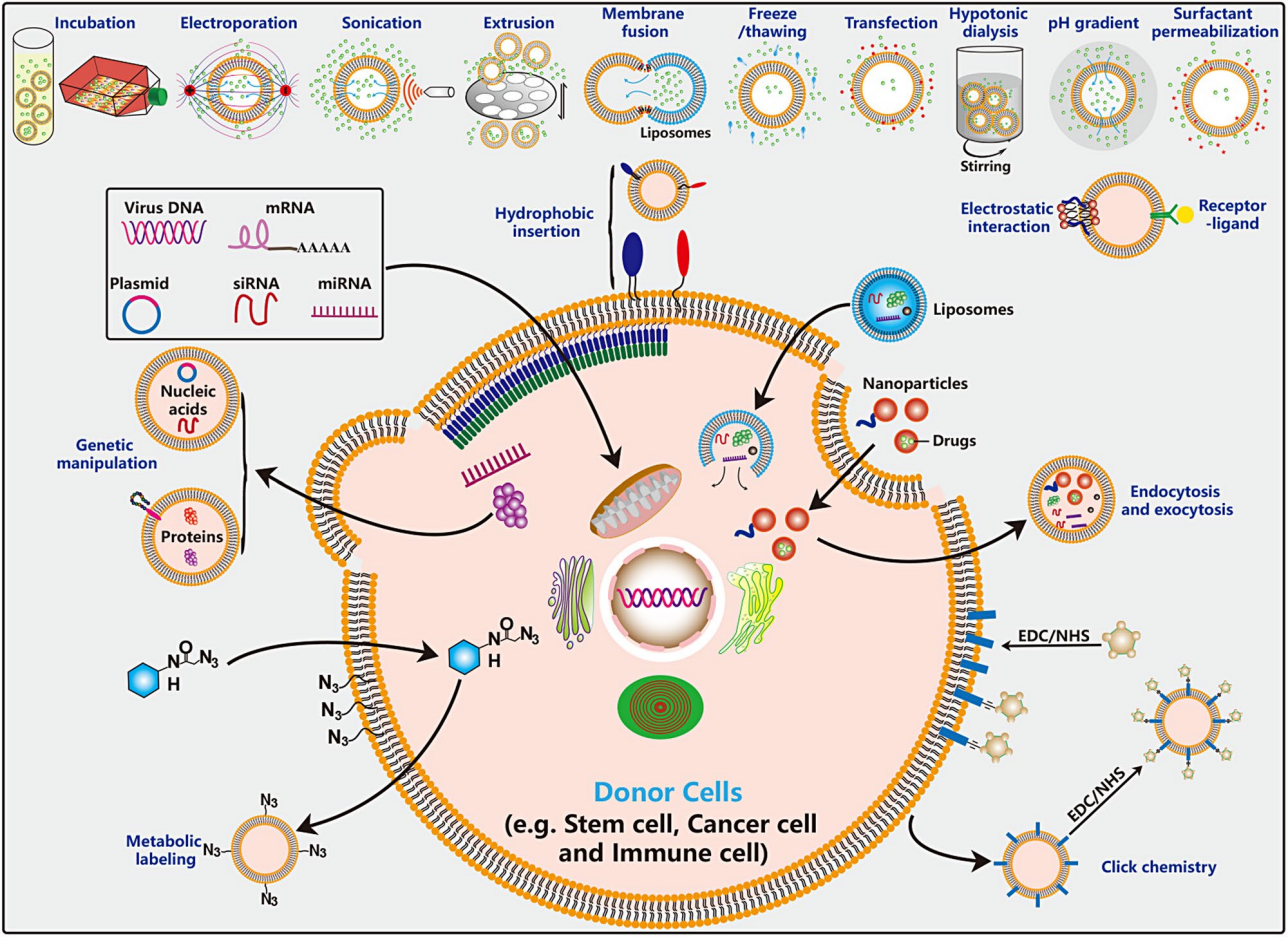
Functionalized approchs of EVs. Traditional bio-techniques and advanced nanotechnologies have been generalized in the engineering modification of EVs. These methods are divided into two main categories: membrane modification and content loading.

The excellent properties of EVs, such as active substance carrier, good biocompatibility, high circulatory stability, low immunogenicity and toxicity and ability to penetrate biological barriers and escape systemic clearance, have aroused great interest among researchers in the field of cancer therapy. There are many sources of EVs, including mammalian cells, bacteria, plants, honey (Chen et al., 2021), and body fluids (Figure 5). Unmodified produced EVs directly as potential anti-tumor drugs are limited (Table 2). Engineered EVs enhances the ability of targeted delivery to tumor sites, and plays a synergistic effect in combination with other therapeutic methods, which obviously has greater application potential (Table 3). Engineered EVs have a broader application prospects and they are gradually becoming a current research hotspot. Loading exogenous antitumor molecules into EVs include passive loading and active loading. Passive loading refers to the antitumor drug molecules and EVs incubation directly or after incubation with donor cells to collect the source EVs. Active load is through the physical methods, such as ultrasonic, extrusion, repeated freezing and thawing, electroporation, chemical methods, such as liposome mediated membrane fusion, click chemistry, biology/cell engineering technology such as gene modification, immunological methods, such as technology, nanotechnology will antitumor molecules are loaded into EVs or connection on its surface (Figure 6).

**Table 3.** The application of functionalized EVs in cancer therapy.

Applica tions	Ligand/EV type	Separation method	EVs sources	Guest molecules	Therapeutic outcomess	Ref
Chemotherapy/targeted chemotherapy	—/sEVs	Centrifugation	MDA-MB-231 cells	Nischarin	Inhibit breast cancer growth	(Maziveyi et al., 2019)
	—/sEVs	Centrifugation&Extrusion	J774A.1 cells	DOX	Target drug delivery	(Rayamajhi et al., 2019)
	—/sEVs	ExoQuick-TC™ Kit	RAW 264.7 cells	PTX/DOX	Chemotherapy for multidrug resistant cancer	(Kim et al., 2016)
	—/MPs	Centrifugation	Human A549 cells	MTX	Target lung cancer chemotherapy	(Guo et al., 2019)
	—/MPs	Centrifugation	Mouse H22 cells	MTX	Drug-delivery in ovarian cancer therapy	(Tang et al., 2012)
	—/MPs	Centrifugation	Human A549 cells	Cis/MTX/DOX	Reverse drug resistance of cancer cells	(Ma et al., 2016)
	iRGD-sEVs	Centrifugation	Mouse iDCs	DOX	Target tumor therapy	(Tian et al., 2014)
	Sgc8-sEVs	Ultracentrifugation	iDCs	DOX	Cancer-targeted chemotherapy	(Zou et al., 2019)
	C-Met-sEVs	Centrifugation &filtration	Macrophage	DOX	Target chemotherapy of TNBC	(Li et al., 2020)
	cRGD-sEVs	Ultracentrifugation	ESCs	PTX	Delivery vehicles for glioblastoma therapy	(Zhu et al., 2019)
	LA-SAV-sEVs	Ultracentrifugation	B16BL6 cells	CpG DNA	Enhance tumor immunotherapy	(Morishita et al., 2016)
	A33Ab-US-sEVs	Ultracentrifugation	LIM1215 cells	DOX	Target colorectal cancer	(Li et al., 2018)
	lipHA-sEVs	Ultracentrifugation	HEK293T cells	DOX	Reverse breast cancer drug resistance	(Liu et al., 2019)
	SPION-sEVs	Magnetic separation	Serum	DOX	Inhibit HCC growth	(Qi et al., 2016)
	SPION-sEVs	Magnetic separation	Serum	DOX	Inhibit HCC growth	(Yang et al., 2021)
	Avidin-biotin-sEVs	Microfluidic chip	HUVECs	PTX	Target drug delivery	(Wang et al., 2017)
	AA-PEG-sEVs	ExoQuick-TC™ Kit	RAW 264.7 cells	PTX	Targeted treatment of pulmonary metastases	(Kim et al., 2018)
	KLA-LDL/LDL-EVs	Ultracentrifugation	L929 cells	MTX	Treatment of glioblastoma multiforme	(Ye et al., 2018)
	CGKRK-EVs	Ultracentrifugation	Cancer cells	TPZ/PTX	Delivery of chemotherapeutic drugs	(Lee et al., 2016)
	CC8-ELVs	Ultracentrifugation	Plasma	imperialine	Target treatment of NSCLC	(Lin et al., 2019)
Immunotherapy/targeted Immunotherapy	—/AB	Ultracentrifugation	Mouse EL-4 cells	AuNR-CpG	Immunotherapy and PTT of cancer	(Zheng et al., 2020)
	A8 peptide aptamer-sEVs	Ultracentrifugation	Serum/Supernatants	—	Restoring anticancer immune response	(Gobbo et al., 2016)
	ssDNA-SA-FasL-sEVs	Differential Centrifugation	THP1/ J774A.1 cells	—	Enhance tumor immunotherapy	(Yerneni et al., 2019)
	αCD3-αEGFR-sEVs	Differential Centrifugation	HEK293T cells	—	Target breast cancer	(Cheng et al., 2018)
	αCD3-αHER2-sEVs	Ultracentrifugation	HEK293T cells	—	Target breast cancer	(Shi et al., 2020)
	PL-L1-OMVs	Ultracentrifugation	Gram-negative bacteria	—	Cancer immunotherapy	(Li et al., 2020)
	CAR-sEVs	Ultracentrifugation	CAR-T cells	—	Inhibit breast cancer growth	(Yang et al., 2021)
	CAR-sEVs	Ultracentrifugation	CAR-T cells	—	Target CD19^+^ B ALL	(Haque & Vaiselbuh, 2021)
	CAR-sEVs	Ultracentrifugation	CAR-T cells	—	Anti-tumor immunotherapy	(Chen et al., 2020)
	LA-CEA/HER2-sEVs	ExoQuick-TC™ Kit	Breast cancer cells	—	Enhance antitumor effects	(Hartman et al., 2011)
	GPI-EGFR-EVs	Ultrafiltration centrifugation	Neuro2A cells	—	Promote tumor cell targeting	(Kooijmans et al., 2016)
	LA-EGFR-sEVs	Ultracentrifugation	HEK293 cells	—	Target treatment of EGFR tumor cells	(Kooijmans et al., 2018)
Gene/targeted gene therapy	FA-GDENs	Ultracentrifugation	Ginger	Survivin siRNA	Inhibit tumor growth	(Li et al., 2018)
	—/sEVs	ExoQuick-TC™ Kit	HEK293 / SKOV3 cells	CRISPR/Cas9 plasmids	Inhibit cancer cell proliferation	(Kim et al., 2017)
	FAP-NVs	Ultracentrifugation	Tumor cells	—	Promote tumor ferroptosis	(Hu et al., 2021)
	—/EVs	Ultracentrifugation	Red blood cells	ASOs/Cas9 mRNA/RNAs	Deliver RNAs to cancer cells	(Usman et al., 2018)
	—/sEVs	ExoQuick-TC™ Kit	Mouse BMMSCs	miRNA-142-3p inhibitor	Reduce tumorigenicity of breast cancer	(Naseri et al., 2018)
	—/sEVs	Ultracentrifugation	Human fibroblast	KRAS siRNA/shRNA	Target oncogenic KRAS	(Kamerkar et al., 2017)
	—/sEVs	Exosome Isolation kit	BMMSCs	antagomiR-222/223, carboplatin	Stimulate cell dormancy	(Bliss et al., 2016)
	—/sEVs	Ultracentrifugation	NK cells	miRNA-let7a	Dual tumor therapy	(Wang et al., 2019)
	—/MVs	Ultracentrifugation	HEK293T cells	mRNA	Inhibit tumor growth	(Mizrak et al., 2013)
	—/EVs	Ultracentrifugation	Cancer cells	PEI/siRNA complexes	Inhibit prostate cancer growth	(Zhupanyn et al., 2020)
	TNF-α-sEVs	Ultracentrifugation	Cancer Cells	CRISPR / Cas9	Activation of necroptosis	(Gulei & Berindan-Neagoe, 2019)
	CD47-CDX/CREKA-sEVs	ExoQuick-TC™ Kit	MEFs	PTEN mRNA	Inhibit glioma growth	(Yang et al., 2020)
	FA-sEVs	Ultracentrifugation	Milk	siRNAs	Inhibit lung cancer growth	(Aqil et al., 2019)
PPT/PDT or targeted PPT/PDT	FA-sEVs	Ultracentrifugation	HEK293T cells	Survivin siRNA	Inhibit tumor growth	(Zheng et al., 2019)
	ClyA-AffibodyHER2-OMVs	Ultracentrifugation	Escherichia coli	KSP siRNA	Inhibit tumor growth	(Kim et al., 2008)
	Lamp2b-DARPinG3-sEVs	Differential Centrifugation	HEK293T cells	TPD52 siRNA	Target breast cancer	(Limoni et al., 2019)
	PSMA aptamer-EVs	Ultracentrifugation	HEK293T cells	Survivin siRNA	Inhibit prostatecancer xenograft	(Pi et al., 2018)
	Lamp2b-IL3-sEVs	Ultracentrifugation	HEK293T cells	BCR-ABL siRNA	Inhibit CML cell growth	(Bellavia et al., 2017)
	PDGFR-GE11-sEVs	Ultracentrifugation	HEK293T cells	miRNA-let-7a	Target breast cancer	(Ohno et al., 2013)
	CXCR4-EVs	Ultracentrifugation	NSCs	antimiRNA-21, miRNA-100	Improve glioma therapy	(Wang et al., 2021)
	FA-sEVs	Ultracentrifugation	Bovine milk	KRAS siRNA, WT p53 plasmid	Inhibit of lung cancer	(Munagala et al., 2021)
	E3-Aptamer-sEVs	Ultracentrifugation	HEK293T cells	SIRT6 siRNA	Target prostate cancer	(Han et al., 2021)
	—/sEVs	Ultracentrifugation	PANC-1 cells	PAK4 siRNA	Target pancreatic cancer	(Xu et al., 2021)
	IL4R-sEVs	Ultracentrifugation	M1 macrophage	NF-κB p50 siRNA, miR-511-3p	Remodeling the antitumor immune microenvironment	(Gunassekaran et al., 2021)
	—/sEVs	Ultracentrifugation	urinary	PMA/Au-BSA@Ce6	Enhance targeted PDT and imaging	(Pan et al., 2020)
	—/sEVs	Exosome isolation reagent	4T1 cells	AIEgens	Enhanced PDT	(Zhu et al., 2020)
	—/Theranosomes	Magnetic sorting	human THP-1 cells	MagNPs / m-THPC	Magnetic targeting PDT and imaging	(Silva et al., 2013)
	ChiP-sEVs	ExoQuick-TC™ Kit	Serum	—	Plasma membrane and nucleus targeted PDT	(Cheng et al., 2019)
	RGD-sEVs	Exosome Isolation kit	MCF-7 cells	V2C Qts	Nucleus-target low temperature PTT	(Cao et al., 2019)
	—/EVs	Ultracentrifugation	mMSCs	mTHPC	Immune reprogramming precision PDT	(Pinto et al., 2021)
Radio-therapy Radio-therapy	—/sEVs	Ultracentrifugation	MSCs	—	Enhance radiotherapy-induced cell death	(de Araujo Farias et al., 2018)
	—/sEVs	Ultracentrifugation	MSCs	miR-34c	Reverse the radioresistance	(Wan et al., 2020)
	—/sEVs	Exosome separation reagent	Colon cancer cells	Gold nanostars	Target tumor NIR-II thermo-radiotherapy	(Zhu et al., 2020)
Synergistic therapy	—/sEVs	Ultracentrifugation	MDA-MB-231 cells	Nischarin	Reduce breast cancer cell motility and tumor growth	(Maziveyi et al., 2019)
	CP05-HMGN1-sEVs	Ultracentrifugation	Cancer cells	—	Inhibit different tumor	(Zuo et al., 2020)
	—/sEVs	Ultracentrifugation	Bel7402 cells	DOX, PSiNPs	Drug carriers for chemotherapy	(Yong et al., 2019)
	—/sEVs	Ultracentrifugation	Mouse 4 T1 cells	CBSA/S100A4 siRNA	Suppress breast cancer metastasis	(Zhao et al., 2020)
	—/MPs	Centrifugation	Mouse H22 cells	Bi2Se3/DOX	PTT and chemotherapy	(Wang et al., 2020)
	DOX-Heparin @NPs-EVs	Ultracentrifugation	Grapefruit	—	Enhance glioma therapy	(Niu et al., 2021)
	—/EVs	Ultracentrifugation	Ginseng	—	Enhance PD-1 mAb anti-tumor efficacy	(Han et al., 2021)
	—/EVs	Ultracentrifugation	4T1/SKBR3/HepG2 cells	antimiR-21 @GIONs	Cancer molecular imaging and therapy	(Bose et al., 2018)
	NRP-1-sEVs	Ultracentrifugation	Raw264.7 cells	SPION@ Curcumin	Imaging and treatment of glioma	(Jia et al., 2018)
	FA/Biotin-MVs	Ultracentrifugation	Cal 27 cells	siRNA/PTX/SA-QDs	Target therapy toward breast cancer	(Zhu et al., 2017)
	DNA-QDs-sEVs	Ultracentrifugation	M1 macrophage	—	Tumor imaging and therapy	(Fan et al., 2019)
	RGD-OMVs	Ultracentrifugation	Attenuated salmonella	Tegafur-loaded micelles	Enhance tumor immunotherapy	(Chen et al., 2020)
	Fe_3_O_4-_sEVs	Ultracentrifugation	THP-1 cells	miR-21	Target and chemo/gene/photothermal therapy	(Wang et al., 2021)
	—/sEVs	Ultracentrifugation	Urinary	Fe_3_O_4_, DOX	Target chemo-chemody namic prostate cancer	(Pan et al., 2021)
	CD47-sEVs	Ultracentrifugation	HEK293T cells	Erastin, RB	Chemo-photodynamic therapy in HCC	(Du et al., 2021)
	CD47-sEVs	Ultracentrifugation	CT26 cells	Thermosensitive liposomes	Photothermal therapy and cancer immunotherapy	(Cheng et al., 2021)

### 4.1. Chemotherapy

**Figure 7.**
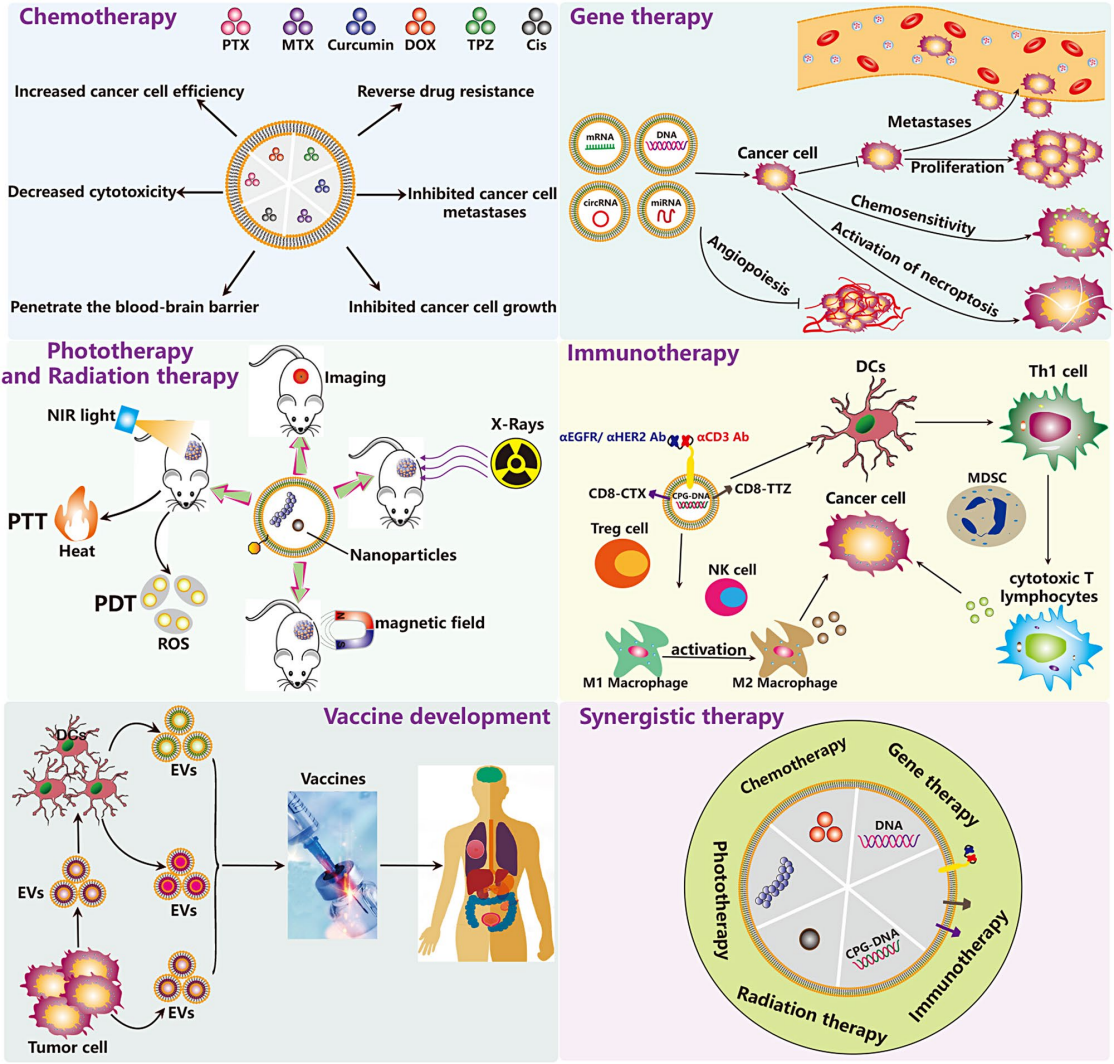
Functionalized EVs for cancer therapy. Currently, the application of Functionalized EVs in cancer therapy has five main directions, including chemotherapy, gene therapy, immunotherapy, phototherapy, and vaccine development.

Chemotherapy is currently one of the main methods for the treatment of malignant tumors. However, due to the lack of specific targeting of tumor sites, anti-cancer drugs have poor efficacy and serious side effects in the clinical treatment process. EVs, as superior drug delivery vehicles, have been shown to deliver a variety of drug molecules to play an anti-tumor role (Figure 7). EVs can be used to deliver various chemotherapeutic drugs such as DOX (Doxorubicin), PTX (Paclitaxel), MTX (Methotrexate), TPZ (Tirapazaming), Cis (Cisplatin), imperialine, and nischarin (Maziveyi et al., 2019). Lung cancer cell-derived EVs enhance the targeted delivery of immunogenic oncolytic adenovirus and PTX in immunocompetent mice (Garofalo et al., 2019). Tumor necrosis factor-related apoptosis-inducing ligand (TRAIL) engineering EVs targeted delivery triptolide for the treatment of malignant melanoma (Jiang et al., 2021). EVs derived from neutrophils have many functions including drug-loading, rapid BBB penetration and enhanced tumor targeting. Wang et al. found that inflammatory tumor microenvironment responsive neutrophil exosomes can be used to delivery DOX for targeted glioma therapy (Wang et al., 2021). Zhou et al. constructed a BMMSC-EV-based galectin-9 siRNA and oxaliplatin (OXA) prodrug dual delivery biosystem for enhancing immunotherapy and reprogramming tumor microenvironment. This combined strategy elicits anti-tumor immunity and achieves significant therapeutic efficacy in PDAC (pancreatic ductal adenocarcinoma) treatment by increasing the polarization of M2 macrophages, recruiting cytotoxic T lymphocytes and downregulating Treg cells (Zhou et al., 2021). Antibody-drug conjugates (ADCs) are a novel class of anti-cancer drugs, which are composed of specifically targeted a monoclonal antibody, a highly effective small molecule cytotoxic drugs, as well as a chemical linker between the two. The surface of EVs derived from cancer cells expresses specific antigen molecules that can bind to ADCs. Therefore, EV-delivered ADCs can be localized at tumor sites or home to the tumor microenvironment to exert anti-cancer effects (Barok et al., 2021). How to effectively enhance the anti-cancer effect of EVs mediated ADCs, and avoid its adverse effects, may be an important direction for researchers to engineer EVs in the future.

### 4.2. Immunotherapy

EVs derived from NK cells have been shown to have good anti-tumor effects. To enhance their efficacy, Zhu et al. used IL-15-pretreat NK cells significantly enhances the anti-tumor potency of NK cell-EVs (Zhu et al., 2019). Engineered EVs showed potential application prospects in cancer immunotherapy and improved therapeutic efficacy (Bell et al., 2016; Yong et al., 2020). To enhance the anti-tumor activity of T cells, the researchers synthesized a multivalent antibody to retarget EVs, which express monoclonal antibodies specific for T-cell CD3, and cancer cell-associated epidermal growth factor receptor (EGFR) (Cheng et al., 2018) or human epidermal growth factor receptor 2 (HER2) (Shi et al., 2020). CpG DNA, a new type of tumor immune activator, can activate innate immune response, enhance antibody-dependent cellular cytotoxicity (ADCC), and can also cause specific immune responses as an effective vaccine adjuvant. Tumor-cell derived EVs anchored assembly CpG-DNA may serve as a promising tumor vaccine through the induction of the cytotoxic T cell response (Matsumoto et al., 2019). The dual-targeting engineered EVs enhance the anti-tumor activity of T cells and the targeting performance of breast cancer. Activating the stimulator of interferon genes (STING) pathway effectively enhances the anti-tumor immune response. However, the rapid clearance and limited cytoplasmic absorption of STING agonists lead to poor pharmacological properties and ineffective targeting, which limits its application in cancer treatment. McAndrews et al. engineered EVs loaded with the STING agonist cyclic GMP-AMP suppressed B16F10 tumor growth and increased accumulation of activated CD8^+^ T-cells and enhanced anti-tumor immunity (McAndrews et al., 2021). B16F10 melanoma cell-EVs expressing TNFSF ligand 4-1BBL and OX40L enhanced anti-tumor immune activity (Semionatto et al., 2020). Shock protein 70 (HSP70) on the membrane of tumor-derived EVs can activate MDSC by interacting with toll-like receptor 2 (TLR2). Gobbo et al. used the A8 peptide to compete with HSP70 on tumor cell EVs to restore the anti-tumor immune response (Gobbo et al., 2016).

Chimeric antigen receptor (CAR) expressed T cells are a powerful and innovative therapeutic strategy for cancer patients. However, problems with serious side effects and toxicities caused by CAR T cells infusion *in vivo* limit its clinical application. Studies have shown that CAR T cells derived EVs have the potential to substitute mother cells for antitumor function. CAR T cell-EVs possess highly effective tumor inhibition rate and low toxicity with no significant side effects *in vitro* and *in vivo* (Fu et al., 2019; Haque & Vaiselbuh, 2021; Yang et al., 2021). Gram-negative bacteria derived outer membrane vesicles (OMVs) containing many pathogen-associated molecular patterns which can activate systemic immune responses. Nanoscale unmodified bacterial OMVs secreted by bacteria are not only a good adjuvant/antigen, but also a superior delivery carrier with great potential in the regulation of tumor immunosuppressive microenvironment. Nevertheless, the consequence of multiple administration of OMVs is a high risk of repeated cytokine storms and systemic coagulation. Recently, it is reported that OMVs derived from attenuated *Salmonella typhimurium* (S.t ΔpG) may be an emerging therapeutic agent for anticancer therapy without obvious adverse responses. Specifically, single intravenous injection of OMVs not only could activate the immune system by boosting the secretion levels of anti-tumor related cytokines, but also could also lead to extravasation of red blood cells in the tumor (Zhuang et al., 2021). Moreover, to overcome the obstacles of antibody-dependent clearance and high toxicity of OMVs when administered intravenously, Qing et al. used a calcium phosphate shell to cover the surface of OMVs, thus achieving effective OMV-based tumor microenvironment reprogramming without side effects (Qing et al., 2020). Engineered OMVs with their enhanced anti-tumor performance and safety have great potential in cancer therapy (Gerritzen et al., 2017; Peng et al., 2020). Compared with the eukaryotic EVs, the bacteria-derived OMVs may be a unique reagent or therapeutic carrier for anti-tumor immunotherapy. Recently, several engineered approaches have been reported to enhance the antitumor immune response of OMVs. Li et al. found that PD-L1 modified OMVs can comprehensively regulate the tumor microenvironment to markedly increase anti-tumor immune efficacy (Li et al., 2020). In addition, Cheng et al. developed a versatile OMV-based antigen display platform to elicit a synergistic antitumor immune response by presenting multiple distinct tumor antigens onto OMV surface (Cheng et al., 2021). Furthermore, Guo et al. designed a sequentially triggered OMVs that loaded with PTX and Redd1 (DNA damage response 1)-siRNA for modulating macrophage metabolism and suppressing tumor metastasis (Guo et al., 2021). Tumor cell (Hu et al., 2021) and macrophage (Mehryab et al., 2020)-derived EVs were hybridized with synthetic liposome for the delivery of DOX for breast cancer targeted therapy.

### 4.3. Gene therapy

Gene therapy is a promising cancer treatment technology, which mainly aims at the tumor genome changes and carries on the corresponding intervention. MiRNA-199a overexpressed-BMMSC-EVs inhibited glioma progression by down-regulating ArfGAP with GTPase domain, ankyrin repeat and PH domain 2 (AGAP2) (Yu et al., 2019). BMMSC-derived EVs loaded with antagomiR-222/223 stimulate cycling quiescence and early breast cancer dormancy (Bliss et al., 2016). MiR-122-modified AD-MSCs derived EVs significantly increased the chemosensitivity of hepatocellular carcinoma cells to sorafenib (Lou et al., 2015). HucMSC-EVs delivered exogenous miR-145-5p to inhibit pancreatic ductal adenocarcinoma progression (Ding et al., 2019). BMMSC-EVs delivered exogenous miRNA-143 to inhibit cell migration and invasion of human prostate cancer by downregulating trefoil factor 3 (TFF3) (Che et al., 2019). BMMSC-EVs delivered synthetic miRNA-124 and miRNA-145 mimics to glioma cells and glioma stem cells and inhibit their cell migration and self-renewal (Lee et al., 2013). BMMSC-EVs with transfected synthetic miRNA-143 significantly inhibited the migration of osteosarcoma cells (Shimbo et al., 2014). HucMSC-EVs deliver miRNA-375 to downregulate ENAH and inhibit the initiation and progression of esophageal squamous cell carcinoma (ESCC) (He et al., 2020). In 2017, Kamerkar et al. engineered EVs to carry Kras^G12D^ siRNAs or shRNAs to achieve direct and specific targeting to oncogenic KRAS in pancreatic cancer (Kamerkar et al., 2017). Subsequently, they prepared and tested this clinical-grade good manufacturing practice standard engineered EVs for pancreatic cancer in a phase I clinical trial (Mendt et al., 2018). Exogenous miR-let-7i and miR-142 modified tumor-derived EVs could increase the survival rate of tumor-bearing mice and induce reduction in tumor growth by promoting DC maturation and T cell activation along with tumor shrinkage. The administration of EVs loaded with miRNAs enhanced the ability of cytotoxic T cells to produce IFN-γ and Granzyme B (Khani et al., 2021). The authors developed a peptidized EV platform to enhance the delivery of anti-apoptotic Bcl-2 antisense oligonucleotide G3139 (antisense phosphorothioate oligodeoxynucleotide to Bcl2) into tumor cells. HepG2 cell-derived EVs are coupled to cell penetrating polypeptides (CPPs) on the surface to enhance the penetrating ability of EVs and assist EVs to load antisense oligonucleotides (ASOs) (Xu et al., 2021). Covalent conjugation of EVs with peptides and nanobodies for targeted therapeutic delivery (Pham et al., 2021), this method may be effective against solid tumors. In brief, these studies suggest that EV-based gene therapy for cancer has great application value. Currently, the FDA has approved some gene therapies for specific cancer diseases (FDA, 2018a, 2018b). However, how to safely and effectively deliver genetic tools to the primary site of a tumor remains a major challenge.

### 4.4. PTT and PDT therapy

Photothermal therapy (PTT) and photodynamic therapy (PDT) are the two most common optical-based therapies. Under near-infrared laser irradiation, nanoparticles with photothermal conversion effect or photosensitizers can generate heat, singlet oxygen or reactive oxygen free radicals to kill tumor cells. Some studies have shown that EVs can be used as a promising delivery vehicle for targeted delivery of phototherapeutic substances, enhanced tissue penetration of tumor tissues, and real-time multimodal imaging *in vivo*. Recent studies suggest that gold nanoparticles, quantum dots (Qts), multifunctional nanoparticles such as gold-carbon dots, gold nanostars, gold-iron oxide nanoparticles (GIONPs), and PMA/Au-BSA@Ce6, etc. can be decorated on the surface or inside of EVs for the early diagnosis and targeted treatment of tumors. The engineered EVs not only exhibit good tumor PTT and PDT effects, but also exhibit good biocompatibility, safety, long-circulation retention and endosomal escape capabilities.

### 4.5. Radiotherapy

Radiotherapy has become one of the main methods in the treatment of malignant tumors. More than 70% of tumor patients require radiotherapy including comprehensive treatment and individual treatment. Previous research has shown that unmodified EVs combined with radiotherapy have a significant effect on promoting the treatment of tumors (de Araujo Farias et al., 2018). However, radiotherapy resistance is a thorny problem that severely reduces the effect of radiotherapy. Wan et al. demonstrated that miR-34c overexpressing EVs inhibit malignant progression and reverse the radioresistance of nasopharyngeal carcinoma (Wan et al., 2020). In another study, Zuo et al. found that BMMSC-derived EVs alleviate radiation-induced bone loss by restoring the function of recipient BMMSCs and promoting β-catenin expression (Zuo et al., 2019). Multifunctional gold-based nanomaterials are gradually becoming promising candidates for tumor thermal radiation therapy due to their properties in the near-infrared II zone. Recently, Zhu et al. fabricated multifunctional gold nanostars loaded colon cancer cell-derived EVs for penetrative targeting tumor NIR-II thermo-radiotherapy (Zhu et al., 2020). HEK293 cells derived exosomes delivered exogenous miR-22 enhanced the radiosensitivity of ovarian cancer by inhibiting the expression of c-MyC binding protein (MYCBP) and human telomerase reverse transcriptase (hTERT) (Konishi et al., 2020). The above studies showed that EVs have broad prospects in tumor radiotherapy. The rise of engineered EV technology has brought new hopes for enhancing the efficacy of tumor radiotherapy and avoiding its related shortcomings.

### 4.6. Synergistic therapy

There are a variety of anti-tumor treatment methods in clinical practice, but a single treatment method often does not achieve the best therapeutic effect. Therefore, combined treatment methods are often used. Combined treatment can enhance the therapeutic effect while reducing the toxic side effects of drugs, and it can alleviate the suffering of patients. In the treatment of different tumor diseases, the combined application of two or more strategies such as radiotherapy, chemotherapy, immunotherapy, gene therapy and optical therapy is expected to open up new horizons for tumor treatment. EVs were developed for the simultaneous delivery of the anticancer drug 5-fluorouracil (5-FU) and miR-21 inhibitor oligonucleotide to reverse drug resistance in colon cancer (Liang et al., 2020). Recently, Ding et al. designed for the first time an engineered self-activating photo-EV that cleverly combined immunotherapy, PDT and chemotherapy for synergistic anti-cancer treatment (Ding et al., 2021). Bis [2, 4, 5-trichloro-6-(pentoxycarbonyl) phenyl] oxalate (CPPO), Ce6 and the prodrug aldoxorubicin (Dox-EMCH) were simultaneously loaded into M1 macrophage-derived EVs. Many studies confirmed that M1 macrophage-derived EVs have superior tumor homing ability, can select actively target tumor cells, and M1 macrophage-derived EVs repolarizes M2 into M1 macrophages. The engineered M1 macrophage-derived EVs are administered sequentially to induce chemiluminescence and photodynamic therapy, and mediate the release and activation of Dox-EMCH at the tumor site. Wang et al. designed a versatile EV-based chemo/gene/photothermal therapy plantform for the next-generation precision cancer nanomedicines (Wang et al., 2021). Engineered EVs encapsulating PGM5 antisense RNA 1 and oxaliplatin can reverse drug resistance in colon cancer (Hui et al., 2021).

## 5. Biomimetic EV membrane enveloped nanomaterials for cancer therapy

In 2011, Zhang et al. creatively ‘put on’ the cell membrane of nanoparticles, and proposed a revolutionary ‘bionic nanomedicine’ technology, which solved one of the biggest problems that plagued the nanomedicine community (Fang et al., 2018). Inspired by the success of cell membrane delivery of nanoparticles technology, researchers began to try to use the membrane components of EVs to deliver nanoparticles. EVs coated with high-density nano-raspberry (RB@EVs) effectively inhibited tumor metastasis, and RB@EVs induced extracellular leakage in lung metastatic lesions, and also enhanced T lymphocyte infiltration in metastatic lung cancer models (Shen et al., 2021). Chen et al. also reported that EV membrane coated metal-organic framework nanoparticles for protection and intracellular delivery of functional proteins (Cheng et al., 2018). In order to improve the efficiency of EV membrane biomimetic delivery, researchers have developed a microfluidic ultrasound device to produce EV membrane-coated nanoparticles on a large scale for immune evasion-mediated targeting (Liu et al., 2019). To avoid the safety problem of tumor-derived EVs carrying contents, the isolated EVs are treated with hypotonicity and re-prepared into uniform NVs under the action of mechanical extrusion (Li et al., 2020). Although some challenges of EV membrane coating nanotechnology have been continuously solved, the source of EVs faces the challenge of low yield. Currently, researchers are trying to prepare NVs derived from cell membranes to replace the functions of EVs (Kalimuthu et al., 2018).

## 6. Functionalization strategy of EVs

Unmodified EVs have some disadvantages such as targeted insufficient, poor stability, low delivery efficiency and efficacy. In order to overcome these deficiencies, researchers designed some ideal functionalization strategies for engineered EVs according to specific pathogenic mechanisms and corresponding diseases (Jayasinghe et al., 2021). According to the differences in the antitumor molecules carried by EVs and the different methods of engineering EVs, the application of EVs in tumor therapy can be divided into the following two categories: engineered EVs via cell modification, direct modification of EVs. In this part, the principles of engineering strategy design were briefly summarized.

### 6.1. Genetic manipulation

Genetic manipulation is the earliest and most mature strategy in all the operations based on cell modification to engineer EVs. Among them, HEK293 cells, tumor cells, immune cells, and MSCs are the main cell sources of engineering modified EVs (Wu et al., 2021). This strategy can decorate interesting nucleotides such as DNA, mRNA, miRNA, siRNA, circRNA, lncRNA, as well as proteins and peptides to the inside or surface of EVs. In addition, the surface of EVs highly expresses a variety of transmembrane protein molecules such as Lamp2b, CD9, CD63, PDGFR, and CD47, etc. Therefore, membrane protein fusion strategies are often employed to deliver some target proteins and peptides to the surface or inside of EVs through genetic manipulation.

### 6.2. Hydrophobic insertion

Researchers have developed many amphiphilic polymers, (including DSPE-PEG, DMPE-PEG). These nanomaterial with one end coupled to the substance of interest can be embedded into EVs through hydrophobic insertion. Recently, researchers have been inspired by the accumulation of high levels of cholesterol, neurophospholipids and other components in the lipid bilayer of EVs, and have developed some novel lipidomimetic chain for the delivery of goods to EVs (Liu et al., 2019; Yerneni et al., 2019). Folic acid (FA) receptors, integrin αvβ3 and other molecules are overexpressed on the surface of various tumor cells. Aptamer (Yerneni et al., 2019), FA (Wang et al., 2018; Aqil et al., 2019; Zheng et al., 2019), SH (Wang et al., 2018), RGD (Wang et al., 2017; 2017; Zhu et al., 2017; Antes et al., 2018; Wang et al., 2018; Zheng et al., 2019; Zhu et al., 2019), mesenchymal-epithelial transition factor (c-Met) (Li et al., 2020), hyaluronic acid (HA) (Liu et al., 2019), etc., can be anchored on the surface of EVs, and target drugs to specific tumor cells through ligand interactions. Hydrophobic insertion is a simple and common modification strategy via co-incubation. Therefore, it has broad prospects in the application of engineered EVs.

### 6.3. Physical strategies

Co-incubation, electroporation, ultrasonic, extrusion, repeated freezing and thawing, are mainly based on the fluidity of the lipid bilayer of EVs (Lu et al., 2018; Ou et al., 2021). Hypotonic dialysis, PH gradient are mainly based on concentration and osmotic pressure dependence. The co-incubation method is relatively simple to operate and does not destroy the integrity of the lipid bilayer membrane of the EVs. However, it is worth noting that the loading efficiency of this method is low. The efficiency of drug loading into EVs depends on the hydrophobicity of the drugs. Hydrophobic drugs can interact with the lipid membrane of EVs to facilitate their entry into EVs. Electroporation and ultrasound are the most commonly used drug delivery methods with high loading efficiency. They change the permeability of the lipid bilayer membrane of EVs to actively load drugs into EVs. Mechanical extrusion methods are also frequently used to load drugs into EVs, which deform/remodel the EVs through physical extrusion, destroy the EV membrane and mix vigorously with the loaded drug. Freeze-thaw methods temporarily disrupt EV lipid bilayer membranes by forming ice crystals. Freeze-thaw cycles lead to changes in the structure and function of the membrane by removing water molecules from the hydrophilic surface of the plasma membrane. The loading efficiency of other methods is higher than that of co-incubation and lower than that of ultrasound and electroporation (Wu et al., 2021). Similar to the uptake of exogenous materials by cells, the efficiency of uptake of exogenous materials by EVs mainly depends on the characteristics of the materials. For example, glucose-coated nanoparticles can enter the interior of EVs mediated by the GLUT1 protein on the surface (Betzer et al., 2017).

### 6.4. Chemical strategies

Strategies such as chemical reagent transfection and liposome-mediated membrane fusion (Mehryab et al., 2020; Hu et al., 2021; Ou et al., 2021) are mainly to enhance the distance and affinity between the cell membrane or EVs and the delivered molecule under the action of chemical reagents, thereby promoting the effective delivery of substances. The surface saponin penetration strategy uses chemical reagents to change the permeability of the cell membrane to enhance the entry of molecules into EVs (Podolak et al., 2010; Fuhrmann et al., 2015; Haney et al., 2015). Although these methods can achieve the ideal delivery of EV cargo, the remaining chemical reagents such as cationic liposomes, PEG or saponin may be a potential safety threat.

### 6.5. Exogenous material uptake

Exogenous materials such as organic or inorganic nanoparticles can be encapsulated into EVs after ingested by cells. This process is mainly mediated by cell endocytosis and pinocytosis. The efficiency of cell encapsulation of these nanoparticles also depends on nanoparticle size, shape, surface charge, and surface functionality (Wong & Wright, 2016; Zhang et al., 2020), concentrations and incubation times (Correia Carreira et al., 2016). The appropriate size of nanoparticles, cationic materials, PEG modification, high concentration and extended incubation time can all improve the cell uptake efficiency. In addition, the efficiency of cell encapsulation of these nanoparticles also depends on the cell type and size (Wang et al., 2016). Compared with other types of cells, macrophages, as a unmodified phagocytic immune cell, are easier to swallow nanoparticles. In addition, the nanoparticles encapsulated by macrophage-derived EVs can also mediate their escape from the retention of the mononuclear phagocytic system and achieve a good tumor-targeted delivery function.

### 6.6. Metabolic labeling and click chemistry

Metabolic labeling technology is mainly to label chemical groups on cell surface sugars and glycoproteins. These groups through combined bio-orthogonal chemical reactions become a powerful tool to couple multiple components to the surface of EVs (Lee et al., 2018). Click chemistry reactions are often used to further functionalize metabolically labeled EVs due to their mild, high efficiency and high selectivity (Smyth et al., 2014; Wang et al., 2015).

## 7. Delivery of nucleic acids

### 7.1. DNA

The CRISPR/Cas9 vector, as a functional genome editing system, has been successfully applied to the treatment of some human refractory diseases (Frangoul et al., 2021). Currently, synthetic nanocarriers to delivery DNA plasmids face many challenges, such as low efficiency and poor safety. Therefore, how to achieve effective delivery of DNA plasmids has become the key to the development of this field. Researchers have developed some CRISPR/Cas9 system delivery platforms by using EVs, which achieves efficient in vitro genome manipulation in cancer cells, and as well as various hard-to-transfect cells, including MSCs, iPS cells, myoblasts, and neurons. Lin et al. found that Runx2 or CTNNB1 CRISPR-Cas9 expression vectors can be encapsulated into hybrid EVs through the co-incubation of liposomes, plasmids and EVs (Lin et al., 2018). Gee et al. have developed an EV-based ribonucleoprotein delivery system for packaging of CRISPR-Cas9 protein and sgRNA to induce therapeutic exon skipping in Duchenne muscular dystrophy (DMD) patient iPS cells derived skeletal muscle cells and in mdx mice model (Gee et al., 2020). CRISPR/Cas9-loaded cancer-derived EVs suppress expression of poly (ADP-ribose) polymerase-1 (PARP-1), enhance the chemical sensitivity of ovarian cancer cells to cisplatin and induce cell apoptosis (Kim et al., 2017). To overcome the challenge of cellular selective delivery, following this strategy, Zhuang et al. designed specific cell targeting EVs for delivery CRISPR/Cas9 endonucleases. The surface of EVs loaded with the valency-controlled tetrahedral DNA nanostructures that conjugated with DNA aptamer and cholesterol anchor. This system efficiently promoted the tumor-specific accumulation of the EVs in vitro HepG2 cells and human primary liver cancer-derived organoids, as well as *in vivo* xenograft tumor models. The intracellular delivery of RNP by using TDN1-EVs significantly inhibits tumor growth (Zhuang et al., 2020). To improve genome editing efficiency and safety, Yao et al. developed an engineered EVs platform for ribonucleoprotein delivery. This strategy utilizes RNA aptamer and aptamer-binding protein interactions to enrich RNPs into EVs (Yao et al., 2021). In addition to delivering gene editing tools, the new immune adjuvant CpG-DNA can also be delivered to the surface (Matsumoto et al., 2019) and inside of EVs (Zheng et al., 2020) to exert anti-tumor effects.

### 7.2. mRNAs

With the continuous advancement of mRNA synthesis, modification and delivery technology, the stability, safety and translation efficiency of mRNA have been greatly improved, and it has shown great clinical translational value in the field of tumor treatment. EVs are a good gene therapy vector, and they have good prospects for delivering mRNA of interest. Mizrak et al. reported that for the first time genetically engineered HEK293T cells derived EVs expressing high levels of the suicide gene mRNA and protein-cytosine deaminase (CD) fused to uracil phosphoribosyltransferase (UPRT) to inhibit schwannoma tumor growth (Mizrak et al., 2013). In addition, Erkan et al. also found that EVs uploaded with therapeutic CD-UPRT mRNA/protein is a promising strategy for glioblastoma treatment (Erkan et al., 2017). The continuous resolution of challenges such as the safety, efficiency, method, and yield of mRNA delivery to EVs also brings hope for its future clinical applications. Altanerova et al. used engineered MSC derived EVs to develop a prodrug suicide gene therapy for cancer targeted intracellular (Altanerova et al., 2019). Kojima et al. have developed exosomal transfer into cells devices to consistently deliver therapeutic mRNA into target cells for Parkinson’s disease treatment. Recently, Yang et al. developed a cellular nanoporation for large-scale generation of functional phosphatase and tensin homologue (PTEN) mRNA-encapsulating EVs (Yang et al., 2020). This method not only improves the efficiency of mRNA loading into EVs, but also achieves efficient, customizable production of engineered EVs (Kojima et al., 2018).

### 7.3. siRNAs

The silencing-inducing complex mediated by small interfering RNA (siRNA) can simply and efficiently interfere with abnormally expressed genes in tumors to achieve effective treatment of tumors (Alvarez-Erviti et al., 2011). In 2011, Alvarez-Erviti and colleagues were the first to identify and demonstrate that EVs could be a promising engineered drug delivery platform for the treatment of disease. They developed the delivery of siRNA based on DC derived-EVs expressing fusion of Lam2b and neuron-specific RVG peptide. Targeted EVs have been shown to be effective in delivering *GAPDH* and *BACE1* siRNA to nerve cells and significantly knocking down the targeted molecule’s mRNA and protein in Alzheimer’s disease models (Alvarez-Erviti et al., 2011). To improve the efficiency of siRNA delivery by EVs, the authors designed folate-displaying EVs to mediate cytoplasmic delivery of siRNA by avoiding endosome capture (Zheng et al., 2019). Over the past decade, increasing evidences have shown that EVs of different types of cell origin can deliver different siRNA (such as KRAS (Kamerkar et al., 2017), surviving (Pi et al., 2018; Zhupanyn et al., 2020), KSP (Aqil et al., 2019), S100A4 (Zhao et al., 2020), PLK-1 (Greco et al., 2016), BCR-ABL (Bellavia et al., 2017), etc) to treat various tumor diseases (including pancreatic cancer, prostate cancer, colorectal cancer, lung cancer, breast cancer, bladder cancer, and chronic myelogenous leukemia, etc.).

### 7.4. miRNAs

MiRNAs, can be efficiently wrapped into EVs and delivered to recipient cells to perform bioactive functions, inhibiting target mRNA transcription, translation and promoting its degradation. Multiple studies have shown that the use of EVs to deliver exogenous miRNAs provides a novel approach to miRNA-based cancer therapy in a variety of tumors. Unmodified EVs are enriched with a variety of miRNA molecules (such as miR-9-3P, miR-143, miR-375, miR-199a, miR-146b, miR-100, miR1231, miR-145, miR-126-3p, miR-133b, miR-206, miR-186, and miR-3607-3p), and the presence of these endogenous miRNAs in EVs of different cell (such as MSCs (Katakowski et al., 2013; Takahara et al., 2016; Pakravan et al., 2017; Cai et al., 2019; Che et al., 2019; Shang et al., 2019; Wu et al., 2019; Xu et al., 2019; Yu et al., 2019; He et al., 2020; Zhang et al., 2020), NK cells (Neviani et al., 2019; Sun et al., 2020)) origins shows beneficial therapeutic prospects for various cancer treatment. In order to improve the loading efficiency of miRNA into EVs and enhance the therapeutic efficacy of EVs in cancer therapy, the researchers enriched miRNAs into EVs by different engineering strategies, such as gene overexpression of parental cells (Lou et al., 2015; Yu et al., 2019) or loading exogenous miRNAs (Munoz et al., 2013; Shimbo et al., 2014) (including miRNA mimics and inhibitors). Although the efficacy of siRNAs or miRNAs in EVs for cancer treatment is relatively mature. However, how to on demand design, quickly and efficiently, and produce small nucleotides engineered EVs still need to be further improved.

### 7.5. circRNAs

Circular RNA (circRNA) is a new type of non-coding RNA with functions such as regulation and translation. It has a closed loop structure and exists in a large number of eukaryotic transcriptomes. Yang et al. generated rabies virus glycoprotein (RVG) decorated-EVs to targeted deliver circSCMH1 to ischemia-reperfusion injury of the brain, and engineered RVG-circSCMH1-EVs treatment promotes functional recovery in rodent and nonhuman primate ischemic stroke models (Yang et al., 2020). In another experiment, Chen et al. found normal liver cells derived-EVs transmitted circular RNA hsa_circ_0051443 suppressed hepatocellular carcinoma malignant progression by promoting cell apoptosis and arresting the cell cycle (Chen et al., 2020). In conclusion, with the in-depth studies on the functions of circRNAs in tumors, targeted intervention of circRNAs based on the engineered EV strategy will have a broad prospect for the treatment of tumor diseases. EVs have unique advantages over liposome delivery technology, which has attracted the attention of many pharmaceutical companies. With the development and progress of technology, EV-based gene therapy strategies are undergoing clinical translational application.

## 8. Delivery of proteins and peptides

The application of biological functional enzymes and therapeutic proteins in clinical and pre-clinical treatments can inhibit the occurrence and progression of tumors. However, there are still many challenges in the process of systemic protein administration to treat tumor diseases, including easy degradation, low bioavailability, poor targeting, and short half-life. EVs have been suggested as ideal delivery tools for delivery therapeutic proteins, receptors and ligands, cytokines, monoclonal antibodies, and nanobodies. Hao et al. reported that soluble fms-like tyrosine kinase-1(sFlt-1)-enriched EVs derived from HEK293 cells suppressed the growth of small cell lung cancer by inhibiting endothelial cell migration (Hao et al., 2019). Recently, researchers have developed a strategy of membrane fusion for the delivery of macromolecular proteins into EVs. In addition, peptides also can be decorated on the surface of EVs increases the targeting of EVs to disease lesions through genetic manipulation or physical and chemical modification. In recent years, researchers have tried to modify some disease-specific peptides, such as RGD (Tian et al., 2014; Cao et al., 2019; Zhu et al., 2019; Chen et al., 2020), GE11 (Ohno et al., 2013), and other peptides onto EVs in different ways.

## 9. Delivery of drugs

EV-mediated delivery enhances the targeting of drugs, prolongs the half-life of drugs, improves the aggregation of drugs, and reduces the toxic and side effects of drugs at tumor sites (Pullan et al., 2019). Hydrophobic and hydrophilic small molecule drugs can be integrated into EVs by using different methods. After the hydrophilic anti-tumor drugs include DOX (Tian et al., 2014), Curcumin (Cur) (Sun et al., 2010; Aqil et al., 2017), PTX (Kim et al., 2016), and Rhodamine (Yang et al., 2015)), etc., are incubated with the EVs, they can interact with the lipid bilayer of the EVs and enter the EVs driven by the concentration gradient. However, it is worth noting that the loading efficiency of this simple incubation method is not high (Kim et al., 2016). These strategies such as electroporation, saponins, extrusion and dialysis have significantly improved the loading efficiency of this hydrophobic drug delivery to EVs (Fuhrmann et al., 2015). However, these strategies also have some shortcomings such as causing the aggregation of EVs, destroying the activity and integrity of EVs, and preventing the release of drugs (Luan et al., 2017). In order to further promote the clinical translational application of EV drug delivery, more breakthrough delivery strategies need to be further explored.

## 10. Delivery of nanomaterials

### 10.1. Magnetic nanomaterials

Magnetic nanomaterials can actively target the tumor site under an external magnetic field, which have been widely applied to tumor imaging and therapy. However, the inability of nanoparticles to reach a sufficient concentration at the tumor site is the main obstacle that limits their clinical application. Human MSC-derived EVs delivery iron oxide to target ablation of tumor cells via magnetic hyperthermia (Altanerova et al., 2017). Kang et al. have designed magnetic and targeting EVs as a targeted drug delivery vehicle for hepatoma cancer therapy. Superparamagnetic iron oxide nanoparticles are one of the most commonly used nanomaterials for targeted modification of EVs (Zhuo et al., 2021). Multiple superparamagnetic nanoparticles are anchored to the surface of blood-derived EVs through transferrin (Tf)-Tf receptor interaction (Qi et al., 2016). These strategies overcame the problem of insufficient targeting of drugs for cancer therapy, enhanced cancer targeting, and suppressed tumor growth under an external magnetic field. Furthermore, Silva et al. co-coated magnetic NPs and m-THPC photosensitizer into macrophage-derived NVs to enable them to have magnetic and optical response capabilities to achieve therapeutic and imaging functions (Silva et al., 2013).

### 10.2. Au-based nanomaterials

Gold nanomaterials have unique optical properties, high chemical stability, good biocompatibility and easy functionalization. Gold-based nanoparticles (including gold nanospheres, gold nanorods, gold nanocages, and gold nanowires, etc.) have broad application prospects in a variety of biomedical fields (such as bio-imaging, biosensing, and cancer treatment). Based on the many advantages of EVs, researchers combine different types of gold-based nanomaterials with EVs for tumor treatment. Researchers decorated gold nanorods (Wang et al., 2018) or gold nanoparticles (Zhang et al., 2019) on the surface of EVs encapsulated with DOX for combinatorial chemo-photothermal therapy. In addition, in order to facilitate the direct delivery of gold nanoparticles to EVs. Pan et al. prepared monodisperse ultra-small gold nanoparticles (4-5 nm) and sequentially coated them with amphiphilic polymer, BSA and Ce6. The composite nanoparticles are delivered to EVs derived from the urine of tumor patients for real-time fluorescence imaging and enhanced targeted photodynamic therapy with deep penetration and superior retention behavior in tumor (Pan et al., 2020).

### 10.3. Carbon-based nanomaterials

The most researched carbon-based nanomaterials are carbon nanotubes and graphene. Because of their unique advantages, such as larger surface area, higher strength, electrical properties, optical properties, and the ability to carry drugs in the form of non-covalent bonds, carbon-based nanomaterials have great potential in the field of photothermal treatment of tumors. Cao et al. generated TAT peptides-V_2_ C quantum dots engineered RGD-EVs for nucleus-target low-temperature photothermal therapy and three-modality imaging (including fluorescent imaging, photoacoustic imaging, and magnetic resonance imaging) (Cao et al., 2019). Gold-carbon quantum dots, as a composite nanomaterial, have higher biocompatibility and imaging effects than single carbon quantum dots. Jiang et al. synthesized gold-carbon quantum dots which are used to label cancer cell-derived EVs for *in vivo* real-time imaging (Jiang et al., 2018).

### 10.4. Black phosphorus nanomaterials

Black phosphorus (BP) is an emerging two-dimensional metal-free layered material. Due to its good biocompatibility, BP has been explored for potential biomedical applications. Wang et al. reported that bone-related functional cells derived Matrix vesicles (MVs) embedded with BP and functionalized with cell-specific aptamer for molecular recognition-guided biomineralization (Wang et al., 2019). Furthermore, BP quantum dots (BPQDs) have many advantages, such as ultra-small size, good tissue permeability and biocompatibility, higher photothermal conversion efficiency, low side effects, and high *in vivo* degradability. Therefore, BPQDs are superior PTT and PDT reagents for anti-tumor therapy. Serum derived EVs in tumor-bearing mice treated by hyperthermia expressed an array of patient-specific tumor-associated antigens, and strong immune-regulatory abilities in accelerating DC differentiation and maturation. In another study, Liu et al. developed an immunogenic EV-encapsulated BPQDs nanoparticles as an effective anticancer photo-nanovaccine (Liu et al., 2020).

### 10.5. Near-infrared dye and aggregation-induced emission nanomaterials

The phospholipid bilayer of EVs can be labeled with lipophilic dyes, such as PKH dyes (such as PKH26, PKH67) and carbocyanine dyes (such as DiI, DiD, DiO, and DiR). In addition, EVs can also be labeled with some membrane-permeable compounds (such as CFSE, CFDA, and Calcein-AM). After nearly a decade of efforts and development, aggregation-induced emission (AIE) materials have been developed as a new photosensitizer, which has greatly promoted the development of tumor photodynamic therapy. Zhu et al. designed 4T1 tumor cell-derived EVs loaded with AIE luminogen, which facilitate efficient breast cancer penetration and photodynamic therapy (Zhu et al., 2020). In addition, AIE luminogen has also been reported to exhibit superior labeling efficiency and tracking capability for *in vivo* real-time imaging of EVs (Cao et al., 2019; 2020). Therefore, AIE luminogen is expected to become an ideal photosensitizer and imaging agent for tumor photodynamic therapy.

## 11. Challenges and perspectives

Compared with traditional synthetic nanomaterials, EVs have a natural targeting properties, good biocompatibility, stability, plasticity, and safety (Ullah et al., 2021). Therefore, EVs show great commercial value in drugs delivery, diseases diagnosis and prognosis, multimodal imaging tracking, as well as regenerative medicine. Although substantial breakthroughs have been made in the field of engineered EV-based cancer therapy, there still have several shortcomings and technical challenges that may hinder therapeutic applications of engineered EVs. (1) Isolation and purification of EVs. EVs come from a wide range of sources, such as plants, bacterium, honey, mammalian cells culture supernatants, and body fluids. The complex origin of EVs leads to high inconsistency in purity, yield and safety of the vesicles. Although the separation methods have made great progress, they still have some disadvantages. When carrying out scientific research, researchers should combine their own laboratory conditions, carefully choose the most suitable experimental separation technology, and conduct preliminary verification to ensure that the EVs used for engineering have high purity, yield, biological activity, as well as excellent integrity and safety. (2) Large-scale production of EVs. Researchers often need to use a large number of engineered EVs to evaluate the intervention effects of engineered EVs in pre-clinical animal models. This urgently requires a simple and efficient strategy for the large-scale production of unmodified EVs in the future clinical transformation application process. Although some expansion culture devices have been developed, but their production costs, technical difficulties and other issues are still not well resolved. (3) Cargos loading efficiency of engineered EVs. Whether it is endogenous, exogenous or targeted modification of EVs, the loading efficiency of these methods still has huge shortcomings. In the actual operation of engineered EVs, a great quantity of EVs is often destroyed, biological activity is lost, and loading efficiency is insufficient. These problems are partially resolved, but they are far from enough. (4) A universal integrated engineered EV modification platform. Currently, the engineering strategies of EVs are diverse for different tumor diseases. The difficulty of operation, operation time, cost, and other issues of these strategies are very different. Currently, cellular nanoporation and microfluid-based approaches have been used to rapidly and efficiently load therapeutic nucleic acids into EVs (Yang et al., 2020; Wang et al., 2021). In addition, turbulence promotes the production of high-throughput MTHPC-loaded EVs (Pinto et al., 2021). How to develop a direct, fast, on-demand design, and low-cost engineered EV delivery platform will be one of the focuses of more and more attention in the future. (5) Safety of EVs. EVs have similar components to the cells of origin. Some studies have used EVs derived from tumor cells or tumor-associated cells as engineered delivery vectors. However, these EVs themselves may contain many active molecules that promote tumorigenesis and development. Therefore, the effective prevention of this important threat to tumor growth is intriguing. Therefore, their biosafety, biostability and biocompatibility are the biggest challenge for future clinical translational applications. Such as, the problem of self-toxicity of engineered EVs derived from bacteria; Immune rejection caused by transplantation of allogeneic engineered EVs; Security issues based on genetic modification strategies; The problem of reagent residue caused by modification strategies such as physical chemistry; Cardiovascular problems caused by the accumulation of EVs. In some current research reports, during the application of engineered EVs *in vivo*, many experiments did not conduct safety evaluations or only conducted short-term follow-up evaluations. These shortcomings will seriously hinder the safe application of engineered EVs *in vivo* in the future.

## 12. Conclusions

In the past 30 years, EVs have shown excellent efficacy and transformation prospects in preclinical studies and trials. With the development of materials science and the integration of interdisciplinary disciplines, new breakthroughs have been made in advanced separation technologies, detection platforms and engineering modification strategies for EVs. These EVs include unmodified, engineered, or in combination with other nanomaterials, as a new concept as a cancer treatment is an attractive and promising strategy. Here, the latest advances in the field of EV-based and its applications in the creation and customization of therapeutic nanomaterials, and focus on their underlying design principles. With the continuous breakthrough of EV key technical bottlenecks, engineered EVs will become a promising strategy for the treatment of complex and refractory tumors in clinical practice.

## Data Availability

Not applicable.

## Abbreviations

EVs: extracellular vesicles;
MSCs: mesenchymal stem cells;
DCs: dendritic cells;
EMT: epithelial-mesenchymal transition;
Exos: exosomes;
MVBs: multivesicular bodies;
ILVs: intraluminal vesicles;
ESCRT: endosomal sorting complex required for transport-dependent pathway;
GTPases: rab guanosine triphosphatases;
VPS33B: vacuolar protein sorting protein 33b;
TSAP6: p53-regulated protein tumor suppressor-activated pathway 6;
KIBRA: kidney and brain expressed protein;
SIRT1: Sirtuin1;
ESCs: embryonic stem cells;
hBMMSCs: human bone marrow MSCs;
hMMSCs: human menstrual MSCs;
hucMSCs: human umbilical cord MSCs;
TRA2B: transformer 2β;
ESM1: endothelial cell specific molecule 1;
ADMSCs: adipose MSCs;
MARCKS: myristoylated alanine-rich C-kinase substrate;
NK cells: natural killer cells;
GM-CSF: granulocyte-macrophage colony stimulating factor;
αPD-1: anti-programmed death-1;
AFP: α-fetoprotein;
TAM: tumor-associated macrophages;
FasL: fas/fas ligand;
LFA-1: lymphocyte function-associated antigen;
DNAM1: DNAX accessory molecule-1;
PD-1: programmed cell death protein;
TRAIL: tumor necrosis factor-related apoptosis-inducing ligand;
OXA: oxaliplatin;
PDAC: pancreatic ductal adenocarcinoma;
ADCs: antibody-drug conjugates;
EGFR: epidermal growth factor receptor;
HER2: human epidermal growth factor receptor 2;
ADCC: antibody-dependent cellular cytotoxicity;
STING: stimulator of interferon genes;
TLR2: toll-like receptor 2;
CAR: chimeric antigen receptor;
OMVs: outer membrane vesicles;
Redd1: DNA damage response 1;
AGAP2: ankyrin repeat and PH domain 2;
TFF3: trefoil factor 3;
CPPs: cell penetrating polypeptides;
ASO: santisense oligonucleotides;
Qts: quantum dots;
GIONP: gold-iron oxide nanoparticles;
MYCBP: c-MyC binding protein;
hTERT: human telomerase reverse transcriptase;
5-FU: 5-fluorouracil;
CPPO: bis [2, 4, 5-trichloro-6-(pentoxycarbonyl) phenyl] oxalate;
Dox-EMCH: aldoxorubicin;
FA: folic acid;
c-Met: mesenchymal-epithelial transition factor;
DMD: duchenne muscular dystrophy;
PTEN: phosphatase and tensin homologue;
circRNA: circular RNA;
RVG: rabies virus glycoprotein;
sFlt-1: soluble fms-like tyrosine kinase-1;
Cur: curcumin;
Tf: transferrin;
MVs: matrix vesicles;
BPQDs: BP quantum dots;
NSCs: neural stem cells;
FAP: fibroblast activation protein-α;
mRNA: messenger RNA;
siRNA: small interfering RNA;
miRNA: microRNA;
circRNA: circular RNA;
EDC: 1-Ethyl-3-(3′-dimethylaminopropyl)carbodiimide;
NHS: N-Hydroxysuccinimide;
PTX: paclitaxel;
MTX: methotrexate;
DOX: doxorubicin;
TPZ: tirapazamine;
Cis: cisplatin;
PTT: photothermal therapy;
PDT: photodynamic therapy;
ROS: reactive oxygen species
